# Pleiotropic roles of *Clostridium difficile sin* locus

**DOI:** 10.1371/journal.ppat.1006940

**Published:** 2018-03-12

**Authors:** Brintha Parasumanna Girinathan, Junjun Ou, Bruno Dupuy, Revathi Govind

**Affiliations:** 1 Division of Biology, Kansas State University, Manhattan, KS, United Sates of America; 2 Department of Agronomy, Kansas State University, Manhattan, KS, United Sates of America; 3 Laboratoire Pathogénese des Bactéries Anaérobies, Institut Pasteur, Paris, France; 4 Université Paris Diderot, Sorbonne Paris Cité, Paris, France; University of Texas Medical School at Houston, UNITED STATES

## Abstract

*Clostridium difficile* is the primary cause of nosocomial diarrhea and pseudomembranous colitis. It produces dormant spores, which serve as an infectious vehicle responsible for transmission of the disease and persistence of the organism in the environment. In *Bacillus subtilis*, the *sin* locus coding SinR (113 aa) and SinI (57 aa) is responsible for sporulation inhibition. In *B*. *subtilis*, SinR mainly acts as a repressor of its target genes to control sporulation, biofilm formation, and autolysis. SinI is an inhibitor of SinR, so their interaction determines whether SinR can inhibit its target gene expression. The *C*. *difficile* genome carries two *sinR* homologs in the operon that we named *sinR* and *sinR’*, coding for SinR (112 aa) and SinR’ (105 aa), respectively. In this study, we constructed and characterized *sin* locus mutants in two different *C*. *difficile* strains R20291 and JIR8094, to decipher the locus’s role in *C*. *difficile* physiology. Transcriptome analysis of the *sinRR’* mutants revealed their pleiotropic roles in controlling several pathways including sporulation, toxin production, and motility in *C*. *difficile*. Through various genetic and biochemical experiments, we have shown that SinR can regulate transcription of key regulators in these pathways, which includes *sigD*, *spo0A*, and *codY*. We have found that SinR’ acts as an antagonist to SinR by blocking its repressor activity. Using a hamster model, we have also demonstrated that the *sin* locus is needed for successful *C*. *difficile* infection. This study reveals the *sin* locus as a central link that connects the gene regulatory networks of sporulation, toxin production, and motility; three key pathways that are important for *C*. *difficile* pathogenesis.

## Author summary

In *Bacillus subtilis*, sporulation, competence and biofilm formation are regulated by a pleiotropic regulator called SinR. Two *sinR* homologs are present in *C*. *difficile* genome as an operon and henceforth labeled as *sinR* and *sinR’*. Our detailed investigation revealed that in *C*. *difficile*, the SinR and SinR’ are key master regulators needed for the regulation of several pathways including sporulation, toxin production, and motility.

## Introduction

*Clostridium difficile*, a major nosocomial pathogen, is the causative agent of antibiotic-associated diarrhea and pseudomembranous colitis [1, 2]. Every year, nearly half a million cases of *C*. *difficile* infections (CDI) occur in the United States and result in approximately 14,000 deaths [3]. *C*. *difficile* toxins damage the colonic epithelium, which results in moderate to severe diarrhea [4]. Recent studies have shown that these toxins are essential for *C*. *difficile* pathogenesis [4–7]. Due to the strictly anaerobic nature of the vegetative cell, *C*. *difficile* survives outside the host in the form of dormant spores, which are highly resilient and resistant to most disinfectants. Thus, *C*. *difficile* spores are critical for its host to host transmission and persistence in the hospital environment [8].

*C*. *difficile* Toxins A and B are encoded by the *tcdA* and *tcdB* genes respectively, and their expression is dependent on TcdR, an alternative RNA polymerase sigma factor [9–11]. Environmental stresses, such as alteration of the redox potential, high temperature, or limitation of nutrients like glucose, and biotin, modulate toxin production by influencing the expression of *tcdR* [9–12]. Similar to toxin production, the sporulation pathway in *C*. *difficile* is also known to be influenced by nutrient availability and uptake [13, 14]. The regulators involved in controlling toxin synthesis in response to nutrients are the global regulatory proteins CcpA and CodY [14–18]. Among them, CcpA mediates glucose-dependent toxin gene repression [15, 16], and CodY blocks the transcription of toxin genes during the exponential growth phase of the bacterial culture [17, 18]. Other than affecting toxin production, mutations in *codY* and *ccpA* were also found to affect sporulation [13, 16]. Other genes that are known to influence both toxin production and sporulation include *spo0A*, *sigH*, and *rstA* [19–22]. New evidence suggests that the toxin, motility, and sporulation regulatory networks are linked together in *C*. *difficile* [19, 23, 24]. The sigma factor SigD needed for transcription of the flagellar operon was identified to regulate *tcdR* transcription to influence toxin production [25, 26] positively. Mutations in *spo0A*, *rstA*, and *sigH* also influenced motility along with toxin production and sporulation [19–22]. This study identified that mutation of the *sin* locus in *C*. *difficile* could affect toxin production and sporulation along with motility and thus reports a new regulatory element of this network.

In *Bacillus subtilis*, the *sin* (sporulation inhibitor) locus codes for two proteins SinR and SinI and regulates several genes involved in sporulation, motility, competency, proteolysis, and biofilm formation [27–31]. In this study, we have created *C*. *difficile sin* locus mutants in two different strains. Using RNA-Seq analysis, we compared the transcriptome of the mutants with respective parent strains to identify and assess the transcriptional regulation of *sin* locus coded regulators. Follow up phenotypic analyses and complementation experiments showed that the Sin regulators in *C*. *difficile* are also pleiotropic as in *B*. *subtilis*. Here, their regulatory roles in toxin production, sporulation, and motility were further investigated and discussed.

## Results

### Comparison of *C*. *difficile* and *B*. *subtilis sin* loci

**Fig 1:**
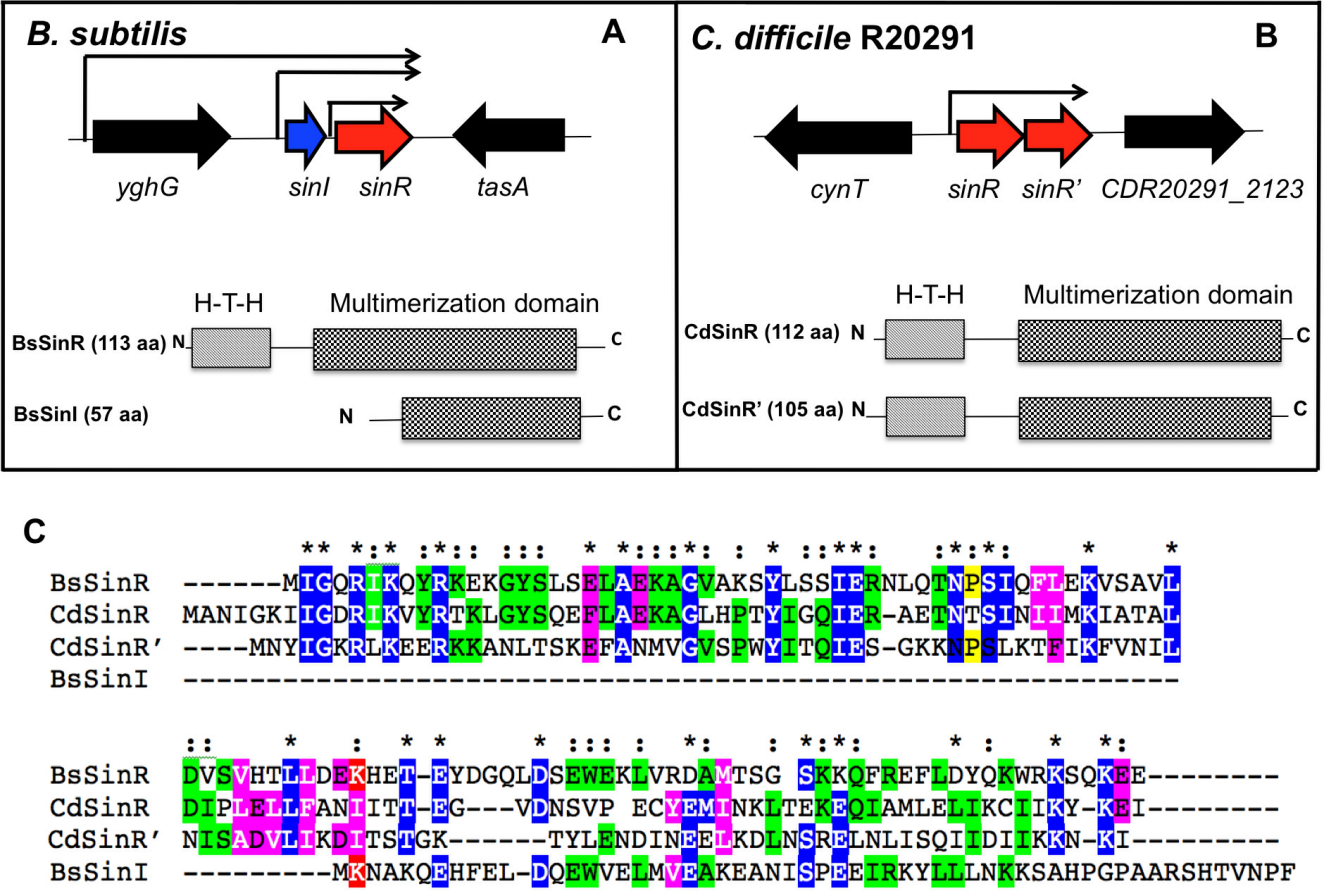
**Genetic organization of genes in the *sin* locus** in *Bacillus subtilis* **(A)** and *C*. *difficile* R20291 strain **(B).** The different domains within Sin proteins are presented below. (C) Sequence alignment of the *C*. *difficile* SinR (CdSinR) and SinR’ (CdSinR’) with *Bacillus subtilis* SinR (BsSinR) and SinI (BsSinI) using ClustalW.

In *B*. *subtilis*, the *sin* locus carries two small ORFs, *sinI* and *sinR* [32, 33] (Fig 1A). *B*. *subtilis* SinR (BsSinR) is a DNA-binding protein that binds to a conserved DNA sequence upstream of the translational start site of target genes to negatively control their transcription. SinI, encoded by a gene adjacent to *sinR*, has an antagonistic relationship with SinR and binds directly to the SinR protein to inhibit its activity. This causes the pathways that were repressed by SinR to switch on. In *B*. *subtilis*, SinR contains 113 aa, and the DNA binding domain is located at the N-terminus part, which spans from residues 5–61 [32, 33] (Fig 1A). The C-terminal part of SinR forms alpha-helices and is responsible for multimerization and SinI interaction. The SinI protein, on the other hand, resembles a truncated SinR without the DNA binding region and carries only the alpha-helical structure to drive the hetero-dimerization of SinR-SinI complex [32–34]. In *C*. *difficile* the *sin* locus contains two ORFs CDR20291_2121 and CDR20291 _2122 (in *C*. *difficile* R20291 reference genome), which codes for proteins that are 43% and 35% identical to *B*. *subtilis* SinR, respectively (Fig 1B). Both these proteins are predicted to be DNA-binding since they carry HTH (Helix-Turn-Helix) domains in their N-terminal regions. Hence we named CDR20291_2121 as *sinR* and CDR20291_ CD2122 as *sinR’*. The *C*. *difficile* SinR (CdSinR) contains 112 amino acids, and its predicted HTH domain spans residues 11 to 66. The SinR’ (CdSinR’) protein carries 105 aa, and its predicted HTH domain spans from residues 7 to 62 (Fig 1B). Both CdSinR and CdSinR’ shows the highest homology to BsSinR in this DNA-binding domain, where within the 50 residues of HTH domain, 13 of them are identical and 19 of them represent conservative substitutions (Fig 1C). CdSinR and CdSinR’ shows similarity with each other (33% identity) only in their N terminal DNA binding domain. The C terminus multimerization domains of these proteins show variations, and there is less similarity of CdSinR and CdSinR’ to BsSinR and each other in this region.

In various *Bacillus sp*. SinR homologs are known to control the expression of the genes adjacent to the *sin* loci. Thus, identifying genes adjacent to the *sin* loci were helpful in predicting at least a few functions of the Sin regulators in these bacterial species. For example, in *B*. *subtilis*, the *sin* locus is adjacent to the *tapA-sipW-tasA* operon, and SinR represses the expression of this operon whose products are involved in the production of the biofilm matrix [31]. In *Bacillus anthracis*, the *sin* locus is next to *calY* that codes for camelysin, a cell surface associated protease, and SinR in this species is known to repress the *calY* expression [35]. In *C*. *difficile*, the *sin* locus is located in between *cynT* (codes for carbonic anhydrase) and CDR20291_2123 (unknown function) (Fig 1B) and is not close to any other genes that are known to be essential for virulence in this pathogen. Thus, the location of the *sin* locus in *C*. *difficile* chromosome did not provide us any clues about its possible functions. To get more information about the locus and its role in *C*. *difficile* physiology we decided to construct and characterize mutants in *sin* locus.

### Construction and verification of *sinRR’* mutants in *C*. *difficile* strains JIR8094 and R20291

An erythromycin resistant marker was introduced in the *sinR* at nucleotide 141 using Clostron, a TargeTron-based group II intron in *C*. *difficile* JIR8094 [36] and R20291 strains [37]. The presence of the retargeted intron in the correct gene in both mutant strains was confirmed by PCR (S1 Fig). In *B*. *subtilis*, three different promoters drive the transcription of the *sin* genes [33]. In *B*. *subtilis*, the polycistronic *sinIR* transcript is produced from two different promoters, and the *sinR* transcript is driven from an independent promoter immediately downstream of *sinI* (Fig 1A) [33]. In *C*. *difficile*, the operon upstream of *sin* locus transcribes in the opposite direction, and no read-through transcription of *sin* locus is possible from its promoter (Fig 1B). Using cDNA prepared from the JIR8094 and the mutant strain, we performed RT-PCR analysis and checked for the presence of *sinR*, *sinR’* and *sinRR’* transcripts (S2 Fig). We could detect *sinR*, *sinR’* and also the read through *sinRR’* transcripts, which confirmed that the *sinR* and *sinR’* are transcribed as a single transcript (S2 Fig). When the same analysis was performed using the mutant strain cDNA both the *sinR*, *sinR’* and *sinRR*’ transcripts were absent (S2 Fig). The QRT-PCR analysis of the *sinR* mutant showed significant reduction of both *sinR* and *sinR’* transcript levels (S2 Fig). It also revealed that similar to *B*. *subtilis*, the *C*. *difficile sin* locus is expressed between late-exponential and early-stationary growth phase (10 to 12 h) (S2 Fig). Similar results were obtained in RT-PCR analyses of cDNA from the R20291 strain (S2 Fig). When we performed the western blot analysis using the SinR and SinR’ specific antibodies (see M&M), both SinR and SinR’ were found to be absent in the mutant (S3 Fig). Our western blot and the RT-PCR results together suggest that *sinR and sinR’* are part of an operon. However, there is a possibility that *sinR’* could have an independent promoter coded within the *sinR* coding region, which was not expressed in the growth conditions tested. Since the insertion of the intron in *sinR* (first gene in the operon) disrupted both *sinR*, *sinR’* transcripts, and SinR, SinR’ production in the growth conditions tested, we named the mutant strains with the disrupted *sinR* gene as JIR8094::*sinRR’* and R20291::*sinRR’*.

### Impact of *sinRR’* inactivation in *C*. *difficile*

We first analyzed the impact of *sin* locus inactivation on the growth of *C*. *difficile* in TY medium. During the exponential phase of the growth, both parents and mutants grew at a similar rate. However, when they entered the stationary phase, we observed a decrease in the turbidity of the mutant cultures as measured as OD@600 nm (S3 Fig). We performed the Triton X-100 autolysis assay to check the influence of SinRR’ on global autolysis of *C*. *difficile* [25]. We used the 16h old stationary phase culture to perform this assay, where the R20291::*sinRR’* lysed at a faster rate compared to the parent (S4 Fig). These results suggested that inactivation of *sinRR’* induced autolysis in *C*. *difficile*. In *B*. *subtilis*, SinR along with another regulatory protein SlrR represses the expression of *lytA-lytB-lytC* and *lytF* autolysins [38]. Our initial observation of lysis phenotype in the *sinRR’* mutants suggested that like *B*. *subtilis* SinR, *C*. *difficile* SinR might also be controlling the autolysin genes. In *B*. *subtilis* the SinR is a pleiotropic regulator and controls various pathways including autolysis [29–31, 33, 38, 39]. We suspected that SinR and SinR’ in *C*. *difficile* might also regulate several targets to control multiple functions. Hence, to identify the *sinRR*’ regulated pathways in *C*. *difficile*, we performed the transcriptome analysis of the *sinRR’* mutants in comparison with their respective parents.

### Assessment of the *sinRR’* regulon in *C*. *difficile*

Based on the growth pattern of the *sinRR’* mutants (S3 Fig) and the expression kinetics of *sinRR’* in the parent strains (S2 Fig), we decided to compare the transcriptomes of mutant strains with their respective parent strains during the early stationary phase (i.e., 12 h of growth) in TY medium. We used three biological replicates and genes were considered differentially expressed if the fold change was ≥ log_2_ 1.5 and their adjusted *p*-value was ≤0.05.

**Fig 2:**
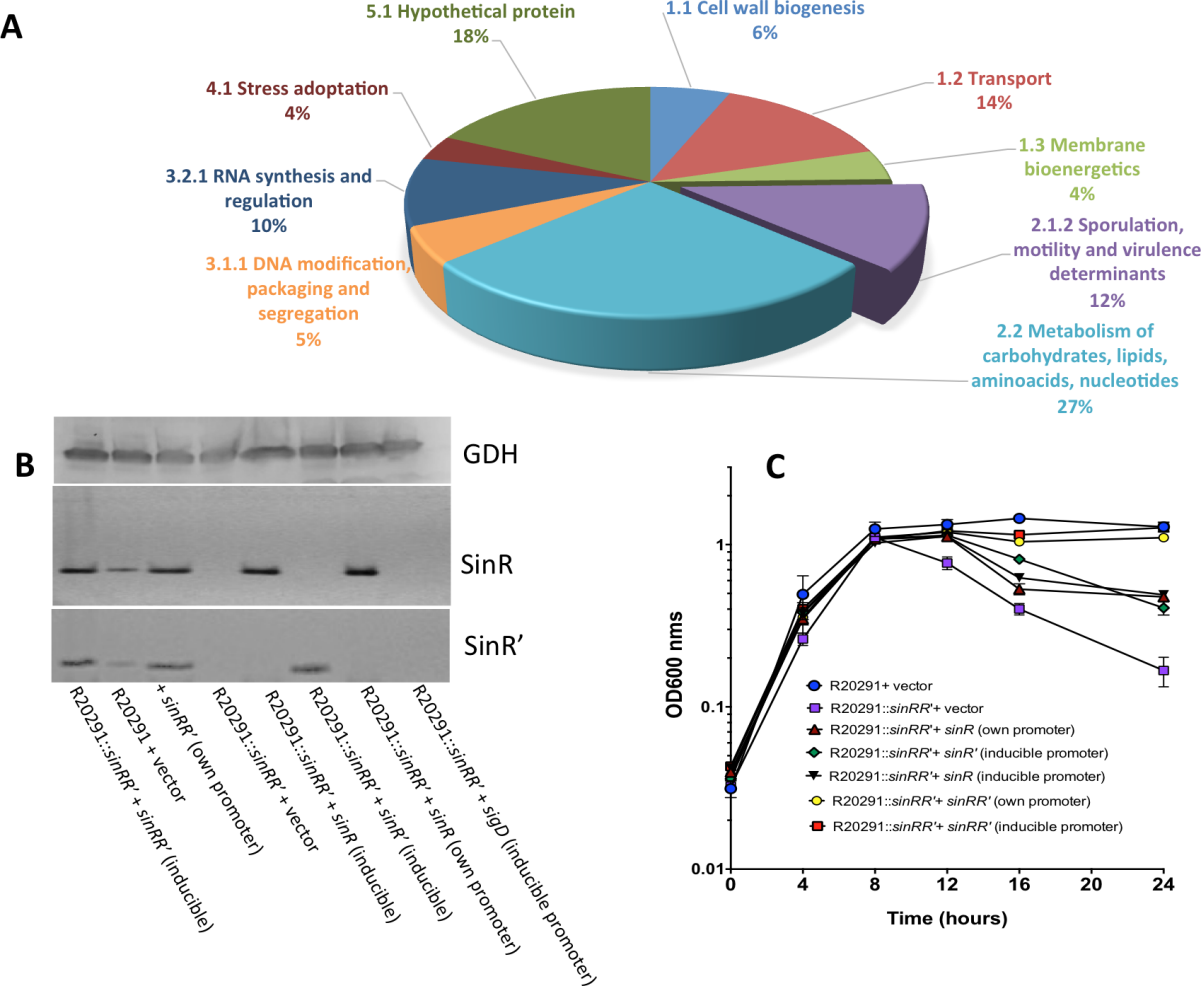
Characterization of *sin* locus (*sinRR’*) mutant in *C*. *difficile*. **(A)** Functional categorization of genes affected by *sin* locus mutation in R20291 strains based on RNA seq data. **(B)** Western blot analysis with SinR and SinR’ specific antibodies demonstrating the absence of both SinR and SinR’ in the *sinRR’* mutants and their presence after the complementation. GDH detection using anti-GDH antibodies was used as loading control. **(C)** Growth curve of the parent (R20291), *sinRR’* mutant and the *sinRR’* mutant complemented strains in TY medium. The data shown are means ± standard errors of three replicates.

In the RNA seq analysis, it was observed that 437 and 425 genes were over-expressed in R20291::*sinRR’* and in JIR8094::*sinRR’* mutant strains, respectively, while 668 and 208 genes were under-expressed in R20291::*sinRR’* and JIR8094::*sinRR’* mutant strains, respectively. Results from the transcriptome analysis confirm that as in *B*. *subtilis*, SinRR’ in *C*. *difficile* also regulates a wide range of genes involved in several pathways including sporulation, motility, metabolism, membrane transport, stress response and toxin synthesis (Fig 2A). A list of genes identified to be differentially regulated in mutants R20291::*sinRR’ and* JIR8094::*sinRR’* compared to their parent strains are listed in S4, S5, S6 and S7 Tables respectively. To test and validate the transcriptome profiles, we performed relevant phenotypic assays and functional analysis with parent and mutant strains for major pathways (sporulation, motility, toxin production and autolysis) that were suggested to be regulated by SinR and SinR’.

We have included following strains in the phenotypic analysis: parent strain, *sinRR’* mutant, *sinRR’* mutant with pRGL311 (plasmid with *sinRR’* under its native promoter), and *sinRR’* mutant with pRG334 (plasmid with *sinRR’* under the inducible promoter). To determine the independent role of SinR and SinR’ in the phenotypes, the *sinRR’* mutant with plasmids: pRG300 (*sinR* gene alone with its promoter region); pRG310 (*sinR* under the inducible promoter); and pRG306 (*sinR’* alone under the inducible promoter) were used. Western blot analysis with SinR and SinR’ specific antibodies were performed to confirm their expressions from the constructs, and the *sinRR’* mutant with vector alone was used as negative controls (Fig 2B). Growth curve analysis showed when *sinRR’* was expressed from its promoter or the inducible promoter in the *sinRR’* mutant, no autolysis was observed, and they grew similar as the wild type (Fig 2C and S4 Fig). In the Triton X-100 autolysis assay, a partial recovery from autolysis was observed when either SinR or SinR’ alone was expressed in the mutant (S4 Fig).

### *C*. *difficile sinRR’* mutants are asporogenic

**Fig 3:**
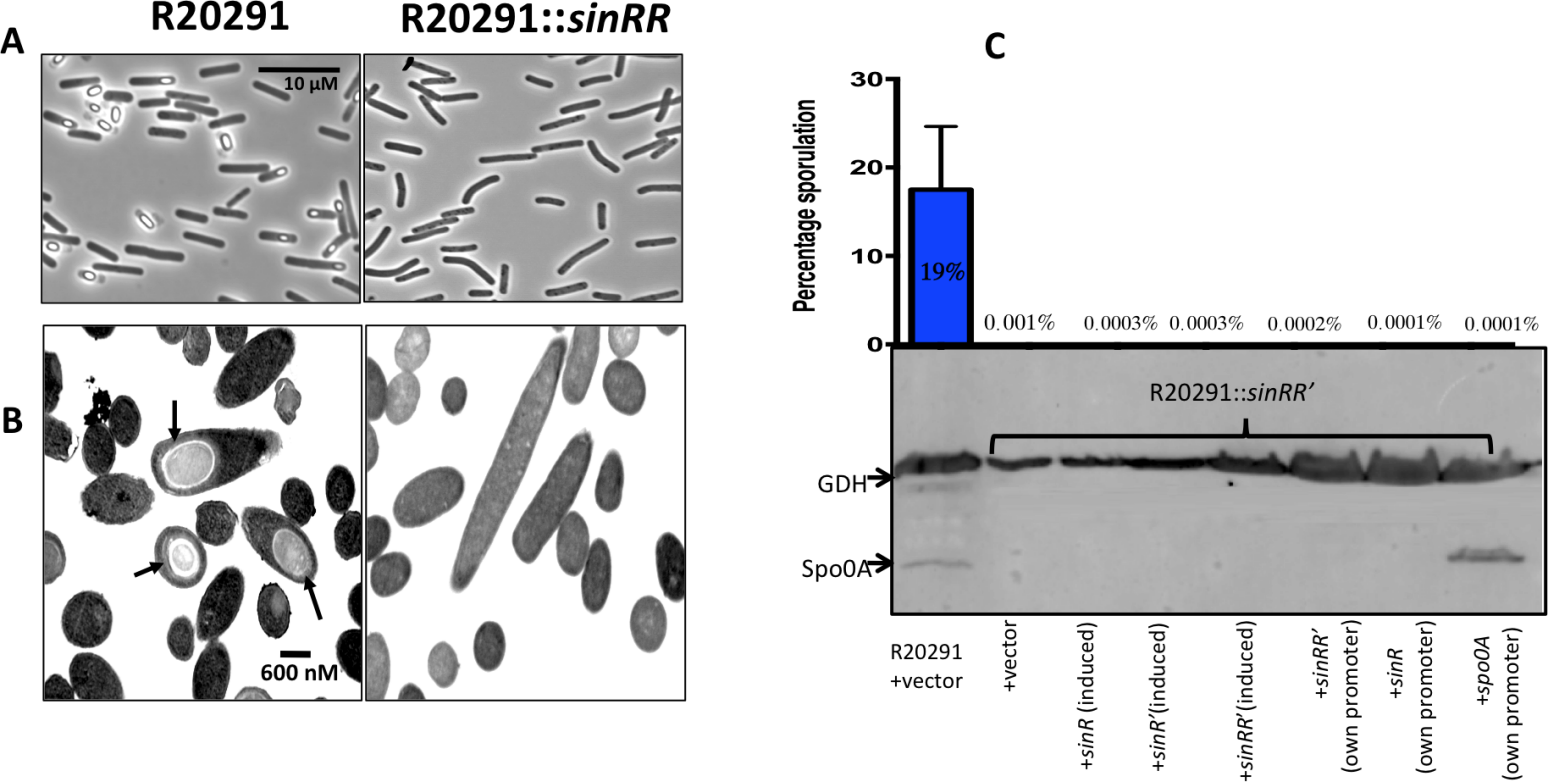
Sporulation in *sinRR’* mutant. **(A)** Phase contrast microscopy of paraformaldehyde-fixed R20291::*sinRR’* strains revealed no spores. **(B)** R20291::*sinRR’* was asporogenic as shown in representative TEM images in comparison with the parent strain. Black arrows indicate mature spores in parent strains. **C.** Asporulation phenotype of *sinRR’* mutant could not be complemented. Sporulation frequency (CFU/ml of ethanol resistant spores) of R20291, *sinRR’* mutant and mutant complemented with different constructs were determined. The *sinRR’* mutant strain expressing *spo0A* from its own promoter was also included in this analysis. Below the sporulation frequency graph is the multiplex-western blot analysis of *sinRR’* mutant complemented strain proteins using Spo0A and GDH specific antibodies.

To determine the role of *sinRR’* on sporulation, we grew the test strains on 70:30 sporulation agar for 30h. Initial analysis through phase contrast microscopy detected no spores in R20291::*sinRR’* (Fig 3A). Transmission electron microscopy (TEM) further confirmed this observation (Fig 3B). Fully mature spores could be detected in R20291, whereas the *sinRR’* mutant cells were devoid of any spores. Similar results were obtained for JIR8094::*sinRR’* mutant as well (S5 Fig).

We performed ethanol treatment based sporulation efficiency assay where the ability of the bacteria to produce viable spores were analyzed by counting the total number of CFU (Colony Forming Units) following ethanol treatment. The mean sporulation efficiency of the parental strain R20291 was 18.7% (Fig 3C). The *sinRR’* mutant strain did not produce any spores, and the percentage of sporulation was near zero. We were surprised by the observation that expression of either *sinRR’* or *sinR*/*sinR’* alone also did not revive the sporulation in the *sinRR’* mutants (Fig 3C).

Sporulation in *C*. *difficile* is initiated with the activation of Spo0A, which in turn triggers early sporulation gene transcription [22, 40]. Transcripts of *spo0A* were 3.5-fold and 2.9-fold under-expressed in JIR8094::*sinRR’* and in R20291::*sinRR’* strains respectively, when compared to parent strains. We performed western blot analysis with the Spo0A specific antibodies [41]. We detected GDH (glutamate dehydrogenase) for loading control since its production was found to be unaffected in the *sinRR’* mutants. Western blot analysis showed that in R20291::*sinRR’* the Spo0A was absent or below the detectable level (Fig 3C, S5 Fig). Lower production of Spo0A can result in down-regulation of all sporulation genes under its control. Our transcriptomic data indeed found many sporulation-associated genes to be affected (Tables 1, S4 and S6) in the *sinRR’* mutant. The QRT-PCR analysis performed on selected sporulation genes confirmed their down-regulation in the *sinRR’* mutants (S8 Table).

**Table 1:** Under-expressed sporulation genes in R20291::*sinRR’*.

Locus Tag	Gene	Protein Name	Fold-Change: R20291/*sinRR’* mutant	log2 ratio	Known/predicted sigma factor needed for expression	Adjusted *p* value
CDR20291_0104	*cwlD*	Germination N-acetylmuramoyl-L-alanine amidase, Autolysin	67.7	6.1	SigE	1.01E-09
CDR20291_0125	*spoIIID*	Stage III sporulation protein D	68.0	6.1	SigE	6.24E-05
CDR20291_0128	* *	putative sporulation protein yyac, DUF1256 family	50.2	5.6	SigE	0.005
CDR20291_0213	* *	hypothetical protein	3041.9	11.6	SigE	6.05E-14
CDR20291_0316	* *	spore coat assembly asparagine rich protein	81.8	6.4	SigE	0.00057
CDR20291_0713	* *	Putative sporulation protein YunB	63.0	6.0	SigE	0.00666
CDR20291_1005	* *	Putative membrane protein, BDBH YlbJ involved in spore cortex formation	5.7	2.5	SigE	5.93E-06
CDR20291_1030	*spoIIIAA*	Stage III sporulation protein AA	18464.8	14.2	SigE	0.00005
CDR20291_1031	*spoIIIAB*	Stage III sporulation protein AB	90.8	6.5	SigE	8.33E-06
CDR20291_1033	*spoIIIAD*	Stage III sporulation protein AD	3571.7	11.8	SigE	1.32E-08
CDR20291_1034	*spoIIIAE*	Stage III sporulation protein AE	13.9	3.8	SigE	1.48E-09
CDR20291_1035	*spoiIIIAF*	Stage III sporulation protein AF	119.5	6.9	SigE	0.00825
CDR20291_1036	*spoIIIAG*	Stage III sporulation protein AG	794.0	9.6	SigE	2.08E-09
CDR20291_1051	*spoIVB*	Stage IV sporualtion protein AB	6.2	2.6	SigE, SigG	6.61E-06
CDR20291_1073	* *	Putative phage protein, skin element	98.9	6.6	SigE	0.00821
CDR20291_1282	*cotE*	Spore coat protein CotE peroxiredoxin/chitinase	18.7	4.2	SigE	0.00029
CDR20291_1360	*cotB*	Spore outer coat layer protein CotB	332.0	8.4	SigE	1.03E-07
CDR20291_2146	*cspC*	Subtilisin-like serine germination related protease- CspC	16.3	4.0	SigE	0.00921
CDR20291_2147	*cspBA*	Subtilisin like serine germination related protease, CspB	19.5	4.3	SigE	7.88E-07
CDR20291_2289	*cotJA*	Putative spore coat protein	103	7.0	SigE	0.00074
CDR20291_2291	*cotD*	Spore coat protein CotD manganese catalase	139.6	7.1	SigE	0.056
CDR20291_2334	*spoIV*	Stage IV sporulation protein	4.1	2.0	SigE	9.981E-06
CDR20291_2335	* *	putative sporulation protein yyac	1593.4	10.6	SigE	0.00097
CDR20291_2513	*spoIVA*	Stage IV sporulation protein AA	309.1	8.3	SigE	2.35E-06
CDR20291_2573	*spoIIE*	Stage II sporulation protein E	36.13938	5.17	SigE	7.04E-05
CDR20291_3331	* *	Putative spore protein	3.1	1.6	SigE	6.75E-09
CDR20291_3376	*spmB*	Spore maturation protein B	41.4	5.4	SigE	0.00081
CDR20291_3377	*spmA*	Spore maturation protein A	21.5	4.4	SigE	2.80E-12
CDR20291_3404	*sipL*	SpoIVA interacting protein	18.3	4.2	SigE	8.71E-05
CDR20291_0124	*spoIIQ*	Stage II sporulation protein Q	56.9	5.8	SigF	0.05
CDR20291_0213	* *	hypothetical protein	3041.9	11.6	SigE, SigF	6.05E-14
CDR20291_0316	* *	spore coat assembly asparagine-rich protein	81.8	6.4	SigE, SigF	0.0005
CDR20291_2362	*spoIIP*	Stage II sporulation protein P	15.1	3.9	SigF	2.67E-11
CDR20291_2363	*gpr*	Spore endopeptidase	28.8	4.8	SigF	1.83E-07
CDR20291_2576	*sspA*	small acid-soluble spore protein A	196.8	7.6	SigG, SigF	5.77E-07
CDR20291_3080	* *	small acid-soluble spore protein	9.0	3.2	SigG, SigF	6.98E-06
CDR20291_3107	*sspB*	small acid-soluble spore protein B	305.5	8.3	SigG, SigE, SigF	5.95E-08
CDR20291_3400	*sleB*	Putative spore cortex-lytic enzyme	14.0	3.8	SigF	5.79E-09
CDR20291_2530	*sigG*	RNA polymease sigma-G factor	44.0	5.5	SigG	2.10E-11
CDR20291_0702	*spoVAC*	Stage V sporulation protein VAC	86.6	6.4	SigG	0.000105
CDR20291_0703	*spoVAD*	Stage V sporualtion protein VAD	84.5	6.4	SigG	0.000161
CDR20291_3080	* *	small acid-soluble spore protein	9.0	3.2	SigG	6.98E-06
CDR20291_3336	*spoVT*	Stage V sporulation protein T	309.1	8.3	SigG	9.59E-10
CDR20291_0476	*sleC*	SleC- spore peptidoglycan hydrolase/ germinant receptor complex	642.4	9.3	SigE, SigK	0.00506
CDR20291_0926	*cdeC*	Cysteine rich exosporium protein	101.6	6.7	SigE, SigF, SigK	1.05E-06
CDR20291_1282	*cotE*	Spore coat protein CotE peroxiredoxin/chitinase	18.7	4.2	SigE, SigK	0.00029
CDR20291_2289	*cotJA*	Putative spore coat protein	103	7.0	SigE, SigK	0.00074
CDR20291_2291	*cotD*	Spore coat protein CotD manganese catalase	139.6	7.1	SigE, SigK	0.056
CDR20291_3090	*bclA2*	exosprium glycoprotein	8.4	3.1	SigE, SigF, SigK	0.00288
CDR20291_3193	*bclA3*	Putative exosporium glycoprotein	13.1	3.7	SigE, SigF, SigK	0.00543

Since our transcriptome analysis and western blot analysis revealed a lower Spo0A in R20291::*sinRR’*, we decided to test whether the asporogenic phenotype of the *sinRR’* mutants is due to the lower production of Spo0A. We expressed *spo0A* from its native promoter (pRGL312) in the R20291::*sinRR’* and production of Spo0A in *sinRR’* mutants was verified through the western blot analysis using Spo0A specific antibodies (Fig 3C) [41]. To our surprise, production of Spo0A in the *sinRR’* mutants did not induce the sporulation in the R20291::*sinRR’* strain (Fig 3C). For sporulation to proceed normally, the Spo0A protein should get activated by phosphorylation [42]. Spo0A~P then acts as a transcriptional activator for many downstream genes in the sporulation pathway that includes sigma factors, the forespore specific *sigF*, and the mother cell-specific *sigE* [22, 40, 42]. We performed QRT-PCR to detect the transcripts of Spo0A~P activated *sigF* and *sigE* genes. We did not observe increases in *sigF* and *sigE* transcript levels in the *spo0A* expressing *sinRR’* mutant when compared to the *sinRR’* mutant with vector alone control. This result suggests that activation of Spo0A to Spo0A~P is affected in the *sinRR’* mutant.

In *Bacillus sp*., the pathway that controls Spo0A phosphorylation is well characterized [43–47]. In *Clostridia*, the components of this phosphorelay are absent, and it has been hypothesized that sporulation-associated sensor kinases may directly phosphorylate the Spo0A for its activation. In *C*. *difficile*, four orphan kinases (CD630_01352, CD630_2492, CD630_01579, and CD630_1949) are present, among which, the CD630_1579 kinase was shown to phosphorylate Spo0A *in vitro*, and the CD630_2492 mutant was found to be less efficient in sporulation [48]. In the transcriptome data, the CD630_1579 and the CD630_ 2492 kinases were to be under-expressed ~1.5-fold and ~3-fold, respectively, in the JIR8094::*sinRR’* mutant. However, their homologs CDR20291_1476 and CDR20291_2385 in the R20291::*sinRR’* were not affected suggesting that these kinases might not be the main reason for Spo0A inactivation in the *sinRR’* mutants. Since the regulatory network of Spo0A activation is largely unknown, there is a possibility that unknown kinases could have been affected in *sinRR’* mutants.

### *C*. *difficile sinRR’* mutants are non-motile

**Fig 4:**
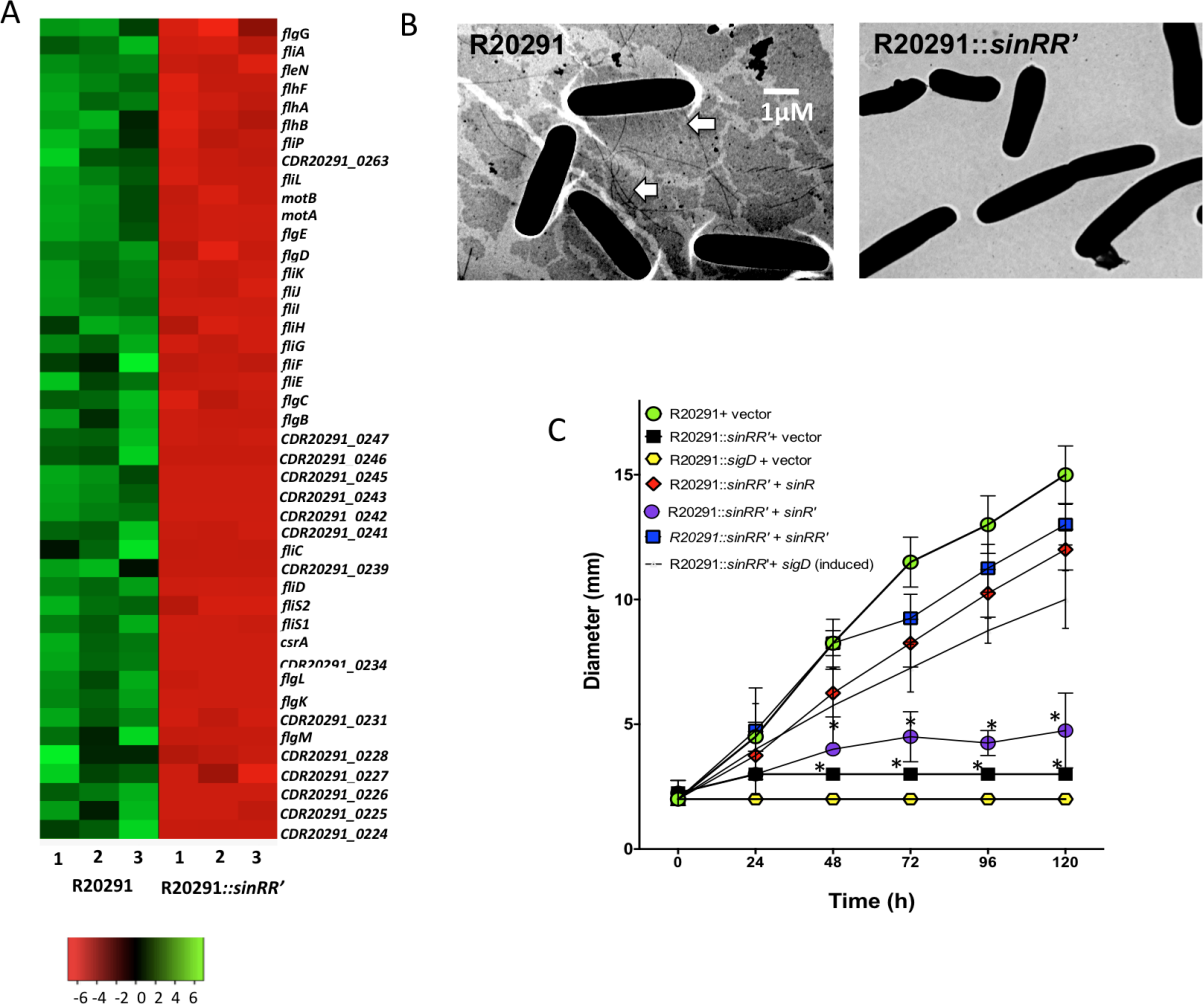
Mutation in the *sin* locus affects *C*. *difficile* flagellar synthesis. **(A)** Heat map showing the lower expression of flagellar and motility-related genes in the R2091::*sinRR’* mutant compared to the parent. Color intensity in each cell represents corresponding Log_2_ expression values in the color scale bar. **(B)** Transmission electron micrographs of negatively stained *C*. *difficile* cells. White arrows point to flagella. **(C)** Motility of R20291, *sinRR’* mutant and the *sinRR’* mutant complemented strains in BHIS with 0.3% agar. The *sigD* mutant and the *sinRR’* mutant expressing *sigD* from an inducible promoter were included in this analysis. The swim diameters (mm) was measured every 24 h for a total of 120 h is shown and the data shown are means ± standard errors of three biological replicates. The experiments were repeated at least three times independently (*, *p*≤0.05 by a two-tailed Student's *t*-test).

The JIR8094 strain was intrinsically non-motile due to mutations within the flagellar operon [49]. Hence, we choose only R20291 and R20291::*sinRR’* to perform motility-related experiments. The R20291::*sigD* mutant and the R20291::*sinRR’* strains with vector alone (pRPF185) were used as the controls. Exponentially growing bacterial cultures were spotted on BHI with 0.3% agar and was incubated at 37°C for 36h to monitor motility. The bacterial cultures expressing *sinRR’*, or *sinR* or *sinR’* from the tet-inducible promoters were spotted on BHI with 50 ng/ml of ATc and 0.3% agar. In the motility assays, the R20291::*sinRR’* strain was defective in motility (Fig 4C and S6 Fig). The transcriptome analysis supported our observation, where *sigD*, the sigma factor needed for the transcription of the flagellar operons, was found to be 14-fold under-expressed in the R20291::*sinRR’* (Fig 4A, S4 Table) along with other motility-related genes. Electron microscopic analysis followed by negative staining failed to detect flagellar structures in the R20291::*sinRR’* (Fig 4B). A dot blot analysis with FliC (the flagellar structural protein) specific antibodies also confirmed the absence of flagella in the R20291::*sinRR’* strain (S6 Fig). Expression of *sinRR’* from its promoter or the inducible promoter revived the motility (Fig 4C). Interestingly, expression of SinR alone was sufficient to revive the motility in the R20291::*sinRR’* strain, whereas the SinR’ expression alone did not have any effect (Fig 4C).

SigD is needed for the transcription of the flagellar operon in *C*. *difficile* [25, 26]. To determine whether the non-motile phenotype of *sinRR’* mutant is due to the reduced levels of *sigD* in the *sinRR’* mutants, we expressed *sigD* from the tetracycline-inducible promoter by introducing the construct pRGL291 into the R20291::*sinRR’* strain (S1). We observed motility was partially restored in the R20291::*sinRR’* when the *sigD* expression was induced (Fig 4C), suggesting that *sinRR’* controls motility by controlling the expression of *sigD* in *C*. *difficile*.

### *C*. *difficile sinRR’* mutants produce less toxins than their parent strains

**Fig 5:**
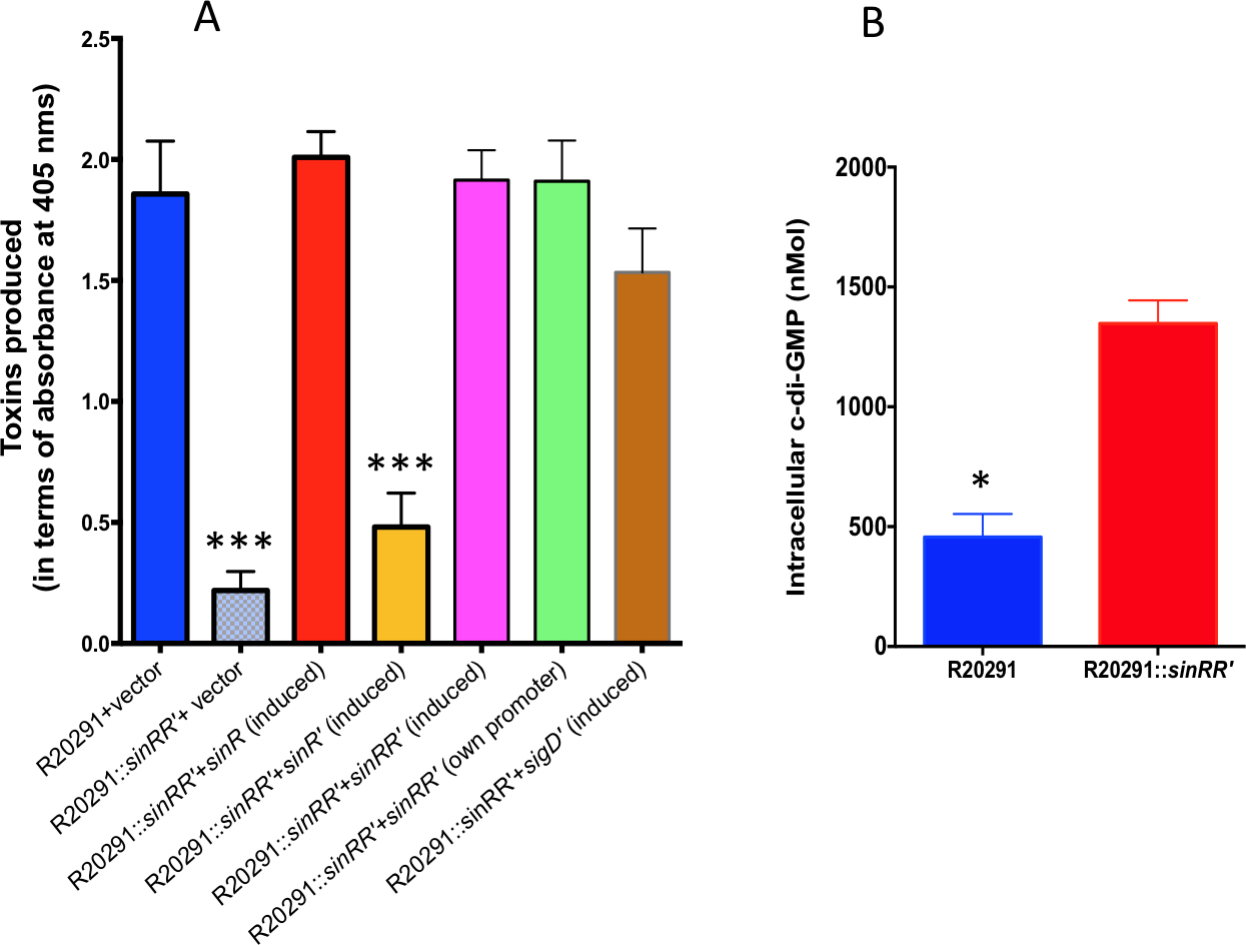
SinRR’ positively influences the expression of PaLoC genes. **(A)** Quantification of toxins in parent R20291 and the *sinRR’* mutant complemented strains using toxins specific ELISA. The data shown are means ± standard errors of three replicates. Statistical significance was tested using one-way ANOVA, followed by Dunnett’s multiple comparisons test comparing values to the average of the parent with vector control (*** <0.0005 *p*-value). **(B)** Increased intracellular levels of c-di-GMP in the *sinRR’* mutant. Statistical analysis was performed using two-tailed *t*-test (* <0.05 *p*-value).

The transcriptome analysis and the follow-up QRT-PCR (Fig 5A, Table 2, S4, S6 and S8 Tables) result suggested *sin* locus’s role in toxin gene regulation. Toxin ELISA was performed with the cytosolic protein extracts of *sinRR’* mutants and their respective parent strains.

**Table 2:** Expression levels of PaLoc genes and their regulators in R20291::*sinRR’*.

Gene	Known or predicted function	RNA-Seq Analysis Fold-change WT/mutant			Q-RT-PCR Analysis Fold-change WT/mutant			Expression in *sinRR’* mutant
		Actual	Log_2_ ratio	Adj.*p* value	Actual	Log_2_ ratio	Adj.*p* value	
*tcdR*	Sigma factor for toxin genes	32.9	5.0	1.11E-16	6.09	2.06	5.35E-03	Under-expressed
*tcdB*	Toxin B	88.4	6.5	5.80E-12	8.02	3.00	1.94E-11	Under-expressed
*tcdE*	Holin like protein	44.2	5.5	6.57E-05	6.29	2.65	3.32E-04	Under-expressed
*tcdA*	Toxin A	13.1	3.7	3.04E-04	7.89	2.98	0.00452	Under-expressed
*sigD*	Sigma factor for flagellar operon	14.4	3.8	4.13E-08	24.76	4.63	6.39E-03	Under-expressed
*dccA*	Diguanylate cyclase	0.10	-3.3	2.05E-24	0.008	-6.93	9.67E-04	Over-expressed
*codY*	GTP sensing transcriptional regulator	0.35	-1.5	7.94E-18	0.12	-3.03	2.23E-05	Over-expressed
*ccpA*	transcriptional regulator	1.29	0.4	3.45E-03	1.95	-0.97	8.45E-08	No significant change

Bacterial cultures expressing either *sinRR’* or *sinR/sinR’* alone from the tetracycline-inducible promoter were grown for 6h in TY medium and were induced with 50ng/ml of ATc for 5 hours. Cytosolic proteins harvested from these induced cultures were used for toxin ELISA. We observed a six-fold reduction in toxin production (Fig 5A) in the R20291::*sinRR’* when compared to the R20291 strain. In JIR8094::*sinRR’* however, a moderate two-fold reduction in toxin level was recorded when compared to the parent strain (S7 Fig). Expression of *sinRR’* in the mutants brought the toxin production back to the level comparable to the parent strains. As we observed in the motility assay, expression of *sinR* alone was sufficient to bring back the toxin production in the *sinRR’* mutant, while expression of *sinR’* did not show any effect. In *C*. *difficile*, SigD positively regulates *tcdR*, the sigma factor needed for toxin gene transcription [25, 26]. Interestingly, the expression of *sigD* from an inducible promoter revived the toxin production in *sinRR’* mutants, suggesting that *sinRR’* controls both toxin production and motility by regulating *sigD* in *C*. *difficile*.

### Elevated c-di-GMP levels are present in *sinRR’* mutant

We observed that SigD expression in the *sinRR’* mutants partially recovered both the motility and the toxin production in that strain (Fig 4C and Fig 5A). The main question that arises from this observation is how SinR controls *sigD* expression. The *sigD* gene is part of the flagellar operon, whose transcription is directly controlled by the intracellular cyclic di-GMP (c-di-GMP) concentration [26, 50]. Within the cells, the c-di-GMP is synthesized from two molecules of GTP by diguanylate cyclases (DGCs) and is hydrolyzed by phosphodiesterases (PDEs) [50, 51]. The functionality of several of these *C*. *difficile* DGCs and PDEs has been confirmed by expressing them heterologously in *Vibrio cholerae*, where they resulted in phenotypes (biofilm formation and motility) that correspond to elevated or lowered levels of intracellular c-di-GMP [51]. In *C*. *difficile* when CD630_1420 (*dccA*) was expressed from an inducible promoter, it resulted in elevated levels of intracellular c-di-GMP and reduced bacterial motility [50]. In R20291::*sinRR’*, ten-fold more (-3.3 Log_2_ fold) *dccA* (CDR2029_1267) transcript was observed (S5 Table) compared to parent. We measured the intracellular concentration of c-di-GMP (S8 Fig) and observed a nearly three-fold increase in the c-di-GMP concentration in the *sinRR’* mutant compared to the parent R20291 strain (Fig 5B). This elevated intracellular level of c-di-GMP in *sinRR’* mutants can block the *sigD* expression, which in turn will result in reduced motility and toxin production (Figs 4C and 5B). Hence, when *sigD* was expressed from the tetracycline-inducible promoter (which is not affected by c-di-GMP concentration), motility and toxin production in the *sinRR’* mutant could be revived. These two findings corroborate our conclusion that elevated levels of c-di-GMP in *sinRR’* mutant plays a major role in controlling its toxin production and motility. We are currently performing experiments to test whether SinR can directly regulate *dccA* in *C*. *difficile*.

### Inactivation of SinR' results in hyper-sporulation, higher toxin production, and motility than the parent strain

**Fig 6:**
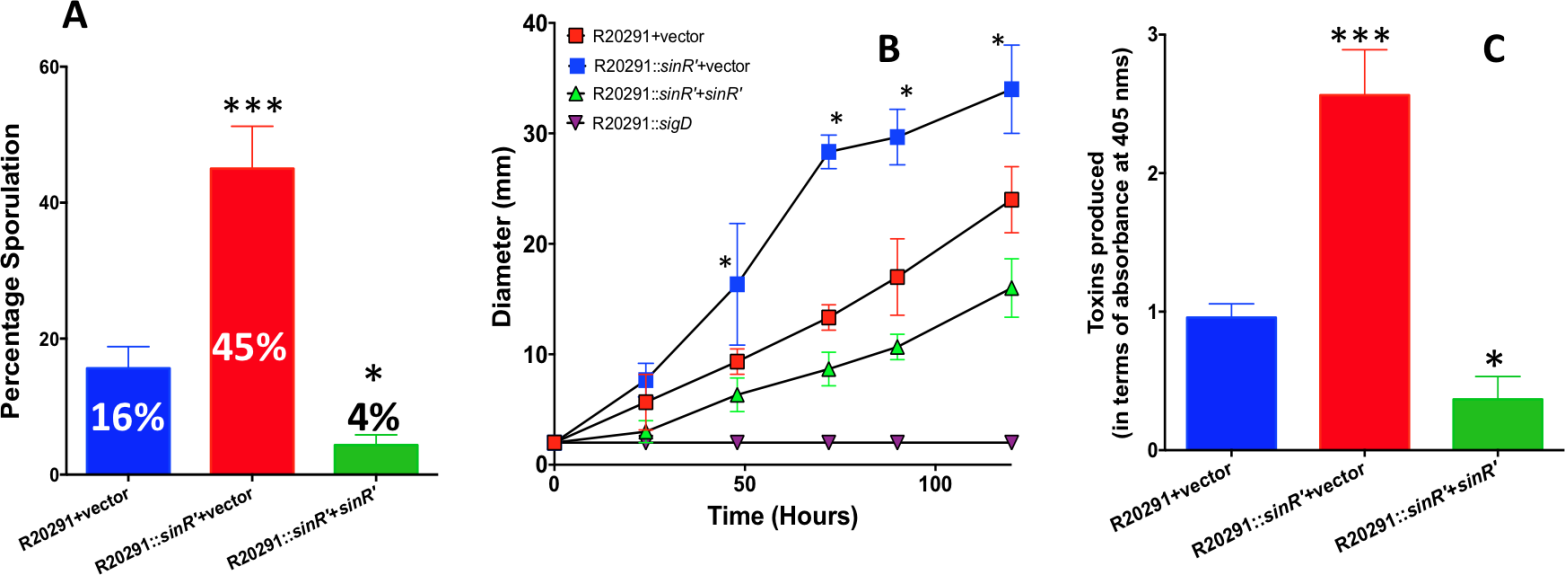
Characterization of *C*. *difficile* R20291::*sinR’*. **(A)** *C*. *difficile* cultures were grown in 70:30 medium for 30 h under anaerobic conditions and Sporulation frequency (CFU/ml of ethanol resistant spores) of R20291, *sinR’* mutant was determined. The data shown are means ± standard errors of three biological replicates. **(B)** Motility assays of the *C*. *difficile* R20291, *sinR’* mutant and complemented *sinR’* mutant. The experiments were repeated at least three times independently (*, *P*≤0.05 by a two-tailed Student's *t*-test). **(C)** Toxin production measured by ELISA. Statistical analysis was performed using one way-ANOVA with Dunnett’s multiple comparisons test comparing values to the average of the parent with vector control (***<0.0005, *< 0.05 *p*-value).

Results from the *sinR* and *sinR’* complementation experiments showed that expression of SinR alone could revive the toxin production and the motility in the R20291::*sinRR’* strain, whereas SinR’ expression alone did not have any effect on the toxin production or the motility (Figs 4C and 5A). These results suggested that among SinR and SinR’, only SinR can directly influence the toxin production and the motility, which raised the question on the role of SinR’ in these pathways. To find the answer, we created a *sinR’* mutant which expressed SinR in the absence of SinR’ (S9 Fig). Our repeated attempts to create a *sinR’* mutant using the similar technique in the JIR8094 background failed for unknown reasons. Mutation in *sinR’* was confirmed by PCR (S9 Fig) and western blot analysis using SinR’ specific antibodies. As expected the SinR’ mutant produced SinR protein, but not the SinR’ (S9 Fig). The R20291::*sinR’* grew almost similar to the parent strain and did not show any profound autolysis phenotype as the R20291::*sinRR’* (S6 Fig). We performed the assays to measure sporulation, motility and toxin production in the R20291::*sinR’*. In the sporulation assay, it was found that R20291::*sinR’* produced nearly three-fold more spores than the parent R20291 strain (Fig 6A). The R20291::*sinR’* was more motile than the R20291 strain (Fig 6B). Similarly, a 2.5-fold increase in the toxin production was observed in the R20291::*sinR’* when compared to the parent strain (Fig 6C). These initial results revealed that SinR’ can negatively influence sporulation, toxin production, and motility. In our complementation of R20291::*sinRR’* we showed that presence of SinR’ alone in the *C*. *difficile* cells in the absence of SinR could not influence either toxin production or the motility (Fig 4C and Fig 5A). Hence, SinR’ must be influencing these pathways through its action on SinR. For example, if SinR’ is an inhibitor of SinR then the absence of SinR’ in the R20291::*sinR’* would result in increased SinR activity, which in turn may result in increased sporulation, toxin production and motility in this strain. To test this hypothesis, we performed two experiments. First, tested the effect of over-expressed SinR in the wild-type strain; Second, we checked for physical interaction of SinR with SinR’ proteins by performing pull-down experiments.

### Overexpression of SinR in the wildtype strain R20291 results in hyper- sporulation and increased the toxin production and motility

**Fig 7:**
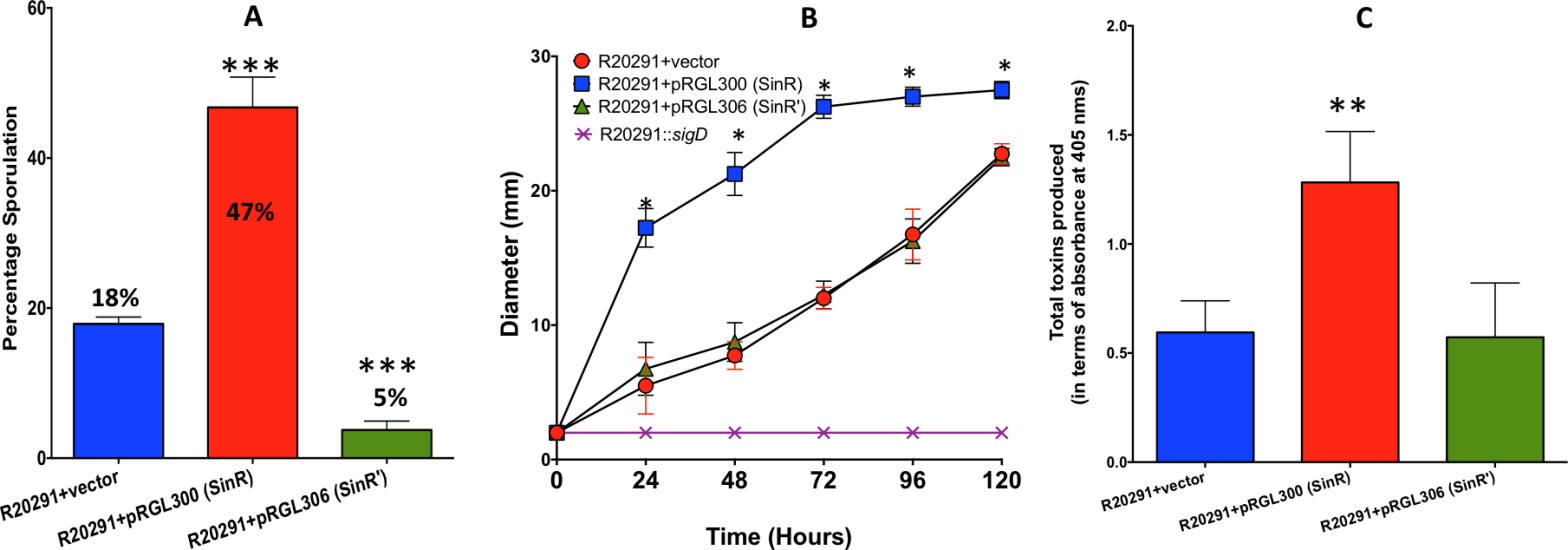
Effect of *sinR* or *sinR’* overexpression in the R20291 strain. The *sinR* or the *sinR’* gene was cloned under tetracycline-inducible promoter and the resulting plasmid constructs were introduced into wildtype (WT) R20291 strain for overexpression. **(A)** Toxin ELISA, **(B)** Motility assay **(C)** Sporulation frequency. The data shown are means ± standard errors of three biological replicates. Statistical analysis was performed using one way-ANOVA with Dunnett’s multiple comparisons test comparing values to the average of the parent with vector control (***<0.0005, *< 0.05 *p*-value).

The plasmid construct with either *sinR* (pRG300) or *sinR’* (pRG306) under tetracycline-inducible promoter were introduced into R20291 parent strain and were tested for their toxin production, sporulation, and motility upon induction with ATc. The R20291 strain with the vector alone was used as the control in these assays. To perform the sporulation assay, we used bacterial cultures grown in 70:30 medium supplemented with 50 ng/ml of ATc for 36 hours. Sporulation efficiency was enumerated as described in the method section. Overexpression of *sinR* in R20291 strain increased its sporulation efficiency 2.5-fold (45%) when compared to the control strain, where the average sporulation efficiency was 18%. Overproduction of SinR’ in R20291, however, reduced the sporulation efficiency to 5% (Fig 7A). Overproduction of SinR in R20291 resulted in increased motility as well (Fig 7B). In *C*. *difficile*, toxin production is minimal during exponential phase (~4 to 8h) of the bacterial culture and reaches its maximum during the stationary phase (12h -16h) [9]. To detect any positive influence of both SinR and SinR’ on toxin production in the parent strain, we chose to use the 8h time point. The bacterial cultures were grown for 6h in TY medium and were induced with 50 ng/ml of ATc for two hours before harvesting their cytosolic protein for Toxin ELISA. Results from these experiments showed that overexpression of *sinR* resulted in a nearly 2.5-fold increase in the toxin production in the R20291 strain when compared to the R20291 with vector alone control (Fig 7C). No significant effect on toxin production was observed when *sinR’* was overexpressed in R20291 (Fig 7C). This could be because *sin* locus is expressed only during the early stationary phase (10-12h) in *C*. *difficile* (S2 Fig). We performed toxin ELISA at 8h time-point when SinR is predicted to be lower in the bacterial cells. If SinR’ acts on toxin production primarily by repressing SinR, then overexpression of SinR’ at this time-point will not have any effect on toxin production. Nevertheless, results from this overexpression studies demonstrated that increased SinR content in *C*. *difficile* could result in increased toxin production, motility, and sporulation.

### SinR’ interacts with SinR

**Fig 8:**
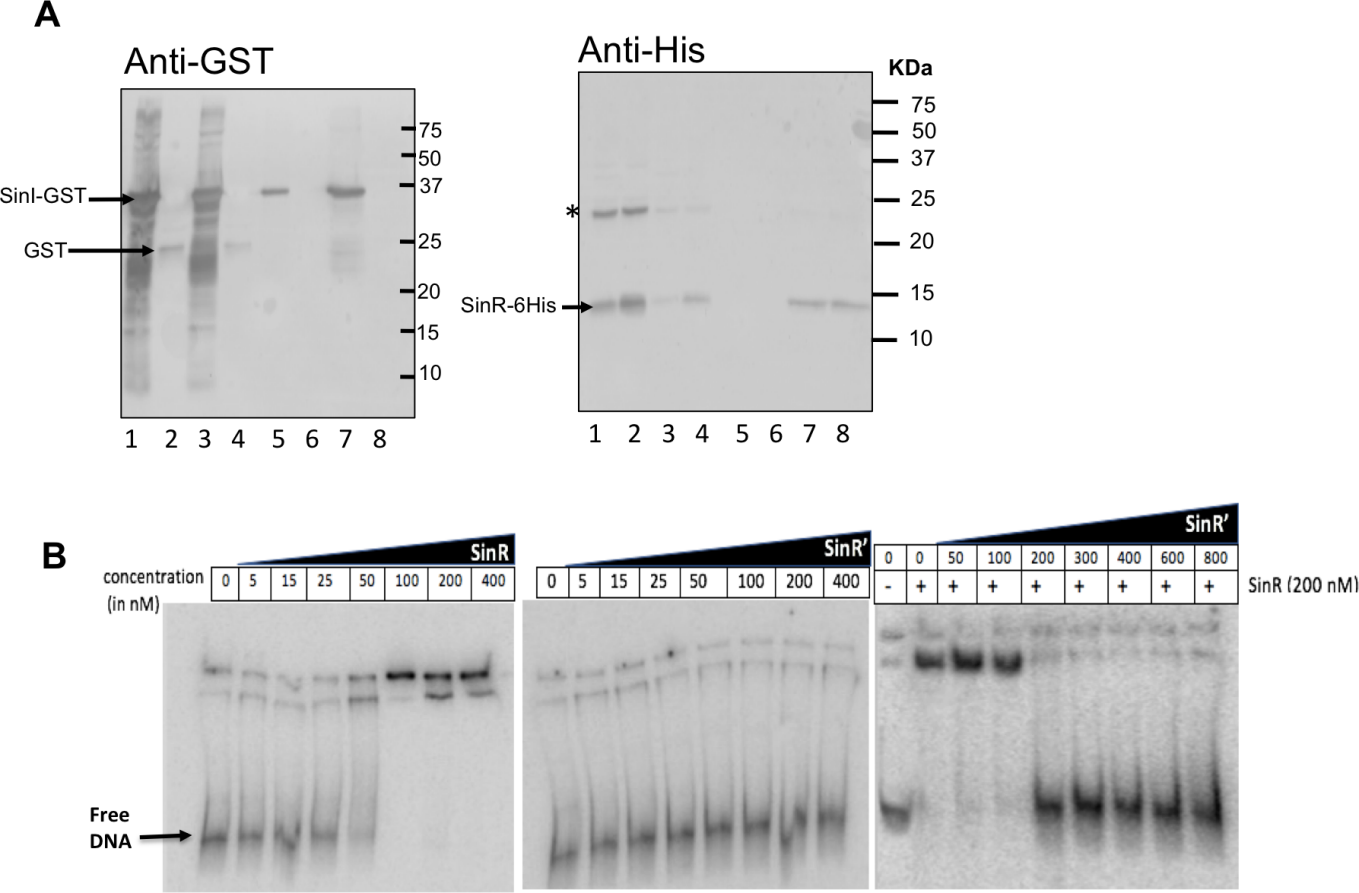
SinR’ interacts with SinR. **(A)** *In vitro*, protein-protein interactions indicate that SinR’ binds tightly to SinR. GST-tagged SinR’ protein was incubated with SinR-6His proteins and purified using Ni++ agarose affinity columns. The elutes were probed with anti-GST and with anti-His antibodies. Lanes details are as follows: Input 1: Mixture of SinR’-GST expressing *E*.*coli* lysate with purified SinR-6His. 1.Input 2: Mixture of GST expressing *E*. *coli* lysate with purified SinR-6His. 2. Unbound from input 1 after passing through Ni^++^ column. 3. Unbound from input 2 after passing through Ni^++^ column. 4. Elute with 50 mm imidazole (SinR’-GST + SinR-6His). 5. Elute with 50 mM imidazole (GST+SinR-6His) 6. Elute with 200 mM imidazole (SinR’-GST + SinR-6His). 7. Elute with 200 mM imidazole (GST+SinR-6His). * indicates SinR-His dimer. **(B)** Interactions of SinR with *codY* promoter region. EMSA analysis of SinR-6His, SinR’-6His, a mixture of SinR’-6His and SinR-His binding to *codY* probe.

In *B*. *subtilis*, SinR monomers bind with each other to form a homotetramer, which would then bind to upstream sequences of the target genes to repress their expression [34, 52]. SinI in *B*. *subtilis* binds with SinR and prevents the SinR homotetramer formation and thus blocks its activity [52]. To test the protein-protein interaction of *C*. *difficile* SinR with SinR’, we performed GST pull-down experiments using SinR-6His and SinR’-GST. Purified SinR-6His protein was mixed with crude lysates from *E*. *coli* expressing SinR’-GST. When we passed this mixture through the Ni^++^ affinity chromatography column, we pulled out SinR-6His along with SinR’-GST, suggesting the tight association of SinR with SinR’ (Fig 8A, lanes 5, 7). In control, the GST alone did not interact with the SinR-6His (Fig 8A, lanes 6, 8), confirming protein specific interaction between SinR with SinR’. These results provided compelling evidence that SinR’ affects toxin production and sporulation indirectly by binding with SinR to inhibit its activity on its target genes.

### SinR binds to *codY* promoter region

Transcriptome analysis of the R20291::*sinRR’* showed up-regulation of *codY*, an important global regulator by ~3 to 30 fold compared to parent strains (S5 Table, S8 Table). CodY is highly conserved in many Gram-positive bacteria [53–55]. In *B*. *subtilis* it regulates several metabolic genes and controls competence, sporulation, and motility [56–58]. In *C*. *difficile*, the *codY* mutant produced more toxins and spores than the parent strains and thus it is a repressor of these pathways [14, 17, 18]. We hypothesized that many phenotypes and transcriptional changes we observe in the *sinRR’* mutant could be related to the up-regulation of *codY* in these mutant strains. To investigate whether SinR and SinR’ or both controls *codY* expression by binding to the promoter region of *codY*, we carried out electrophoretic mobility shift assays (EMSAs). We used radiolabeled DNA probe that contained the putative promoter region of the *codY* gene and performed binding reactions using purified SinR-6His or SinR’-6His proteins. First, we tested SinR alone at increasing concentrations and found that it can shift the probe when used above 100 nM concentration (Fig 7B). When SinR’ was used similarly, it was unable to cause the mobility shift of the probe, even at the highest concentration (Fig 7B). We then tested whether SinR’ would prevent SinR from binding to the *codY* promoter region. To do this, we used increasing amounts of SinR’, in the presence of a fixed amount of SinR (Fig 7B). The results show that the presence of SinR’ in the reaction mix could prevent SinR from binding to the DNA. As a negative control, we used a DNA probe that contained the promoter region of *gluD*, which codes for glutamate dehydrogenase (GDH). Neither SinR nor SinR’ was able to shift the control DNA even at the highest concentrations tested (S10 Fig). Based on these results, we conclude that SinR binds specifically to *codY* promoter region to control its transcription. This result also provided evidence that the SinR’ interaction with SinR prevents its regulatory activity on its target gene.

### CodY regulates *sin* locus expression

**Fig 9:**
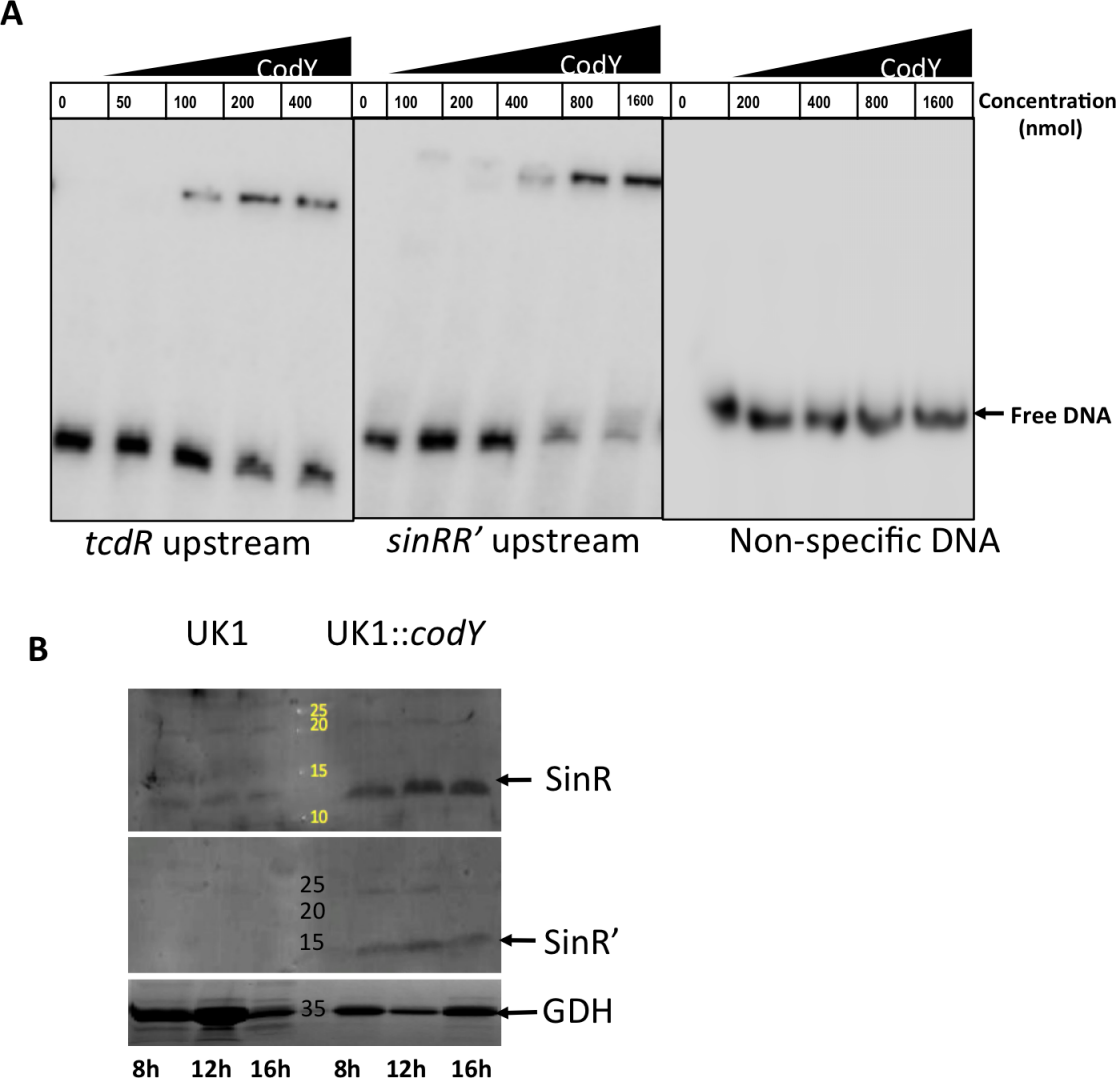
CodY controls the *sin* locus expression. **(A)** CodY-6His binding to *sin* locus promoter region. The *tcdR* upstream and a non-specific DNA probe was as positive and negative controls respectively. **(B)** Western blot analysis of UK1 and UK1::*codY* mutants to detect SinR and SinR’ proteins.

In a recent study, CodY was found to negatively regulate *sinRR’* expression in the *C*. *difficile* 630Δ*erm* strain [14]. A CodY putative binding site was identified in the *sin* locus upstream sequence, and reporter fusions with the *sin* locus promoter revealed the CodY could negatively regulate *sin* locus expression in this strain. However, in the UK1 strain (belongs to the ribotype 027 as R20291), the promoter fusion revealed a positive regulation of *sin* locus by CodY. Because of these contradictory observations, one could not conclude whether CodY regulates *sin* locus. To examine the role of CodY on *sin* locus expression, we performed EMSA with purified CodY-6His and the putative CodY binding region upstream of *sin* locus. An oligonucleotide with putative CodY binding sequence upstream of *sinR* was synthesized (ORG 721) (S2 Table) and was radioactively labeled with [γ- ^32^ P] dATP. A double-stranded DNA probe was generated after annealing with the complementary oligonucleotide (ORG722). It is worth noting no sequence difference was found within this putative *sin* promoter regions of the UK1, R20291, JIR8094 and 630Δ*erm* genomes. We also generated probes with a known CodY binding sequence upstream of the *tcdR* gene (using ORG719 and ORG720) and with non-specific sequence (ORG702 and ORG723) as positive and negative controls respectively. EMSA was performed by incubating the radioactively labeled probes with varying concentrations of purified CodY-6His. We found that CodY could bind to the sequence upstream of *sin* locus at the concentration of 400 nM (Fig 9A). As expected the shift was observed with the positive control probe, while no shift could be observed with the non-specific DNA probe even with high protein concentrations (Fig 9A). Binding of CodY to its targets most of the time results in repression of their transcription [17, 18, 58]. However, there are few targets where CodY was found to promote transcription [58]. To check whether CodY has any positive influence on *sin* locus expression in UK1 strain as reported [14], we performed western blot analysis and looked for SinR and SinR’ in UK1 strain and its *codY* mutant (UK1::*codY*). Results showed that SinR and SinR’ protein content in the UK1::*codY* mutant was higher than in the UK1 parent strain (Fig 9B). Our data demonstrate that CodY has a negative impact on SinR and SinR’ production in this strain. Since our repeated attempts to create *codY* mutants in R20291 and JIR8094 strains failed, we could not include them in this analysis. Nonetheless, our results from the EMSA and the western blot analyses corroborate the negative regulation of the *sin* locus by CodY.

### R20291::*sinRR’* is less virulent in hamster

**Fig 10:**
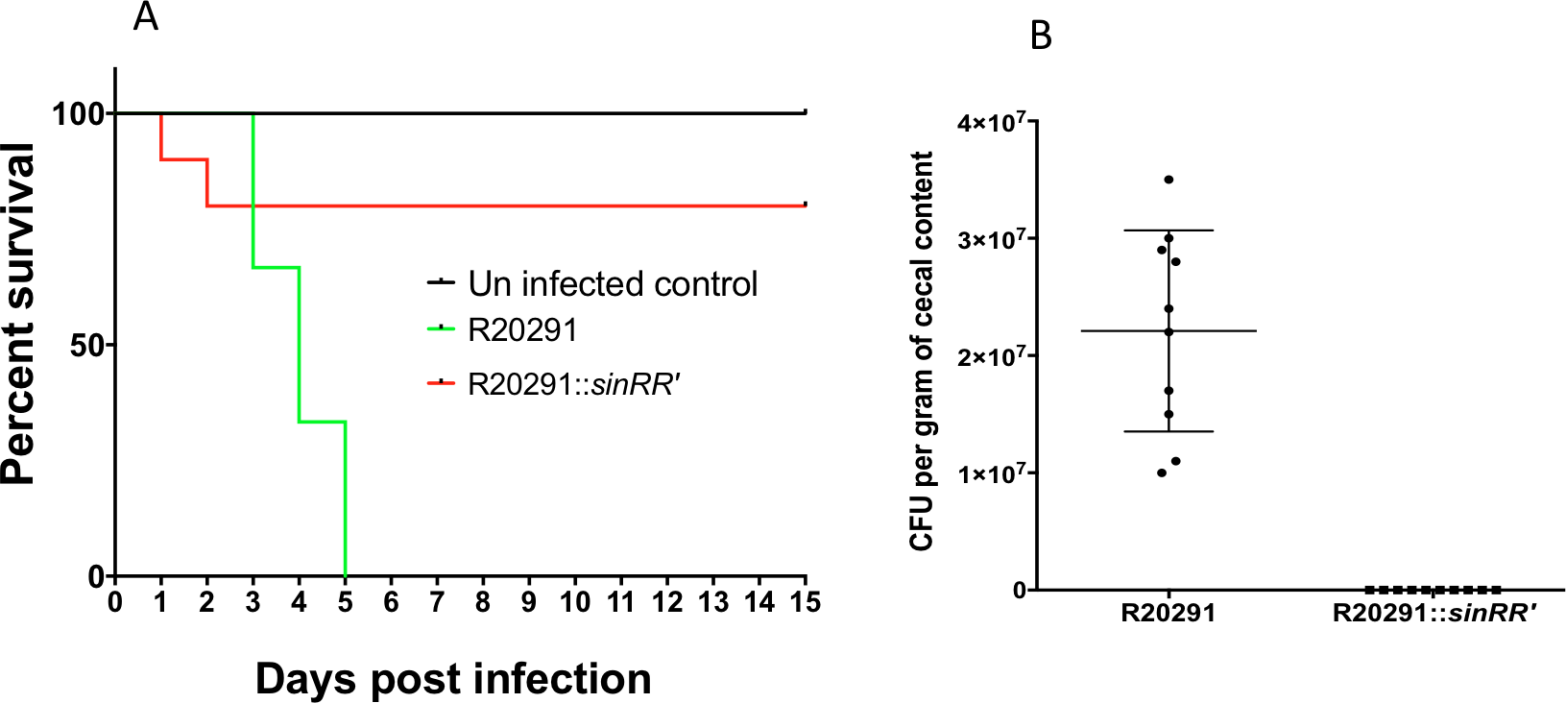
Disrupting *sinRR’* decreases morbidity in hamster models of *C*. *difficile* infection. **(A)** Kaplan-Meier survival curve of clindamycin-treated Syrian golden hamsters inoculated with 2,000 vegetative cells of *C*. *difficile* R20291 (n = 10) or *sinRR’* mutant (n = 10). Six animals were used as an uninfected control. Animals were monitored every four hours for the symptoms of lethargy, poor fur coat, wet tail or hunched posture. Moribund animals were euthanized and *log*-rank statistical analysis was performed; *p*<0.001. (B) Total number of *C*. *difficile* colony forming units (CFU) /gm of cecal contents recovered postmortem.

Since the *C*. *difficile sin* locus was found to be important for the regulation of many important pathways under *in vitro* growth conditions, we wanted to determine its significance in *C*. *difficile* pathogenesis. We used the hamster model in which *C*. *difficile* infection is known to cause severe disease signs [59, 60]. Syrian hamsters were gavaged with 2,000 vegetative cells of *C*. *difficile* strain R20291 or with R20291::*sinRR’* and monitored for *C*. *difficile* infection. Fecal pellets were collected daily until animals developed diarrheal symptoms. All ten animals infected with parental strain R20291 succumbed to the disease within five days after bacterial challenge. Two of the ten animals infected with R20291::*sinRR’* exhibited disease symptoms within two days after challenge (Fig 10A). Diseased hamsters were sacrificed (see M&M), and their cecal contents were collected for toxin ELISA and CFU count. All surviving *sinRR’* mutant infected hamsters (8 in total) and uninfected control hamsters were also sacrificed fifteen days post-infection, and their cecal contents were also tested for toxins and *C*. *difficile* cells. Toxins could be detected (S11 Fig) in the cecal contents of all the diseased hamsters (10 from R20291 group and two from R20291::*sinRR’* group), which confirmed the occurrence of CDI in them. However, toxins could not be detected in the eight hamsters that survived the R20291::*sinRR’* challenge. The cecal contents of R20291 infected hamsters contained nearly 10^7^ colony-forming units per gram. No *C*. *difficile* could be recovered from the cecal contents of any of the R20291::*sinRR’* challenged animals, including of the two hamsters that came down with CDI in this group (Fig 10B). If the *sinRR’* mutant lyses *in vivo*, as we observed in *in vitro* growth conditions, it could explain why we could not recover any *C*. *difficile* cells but could detect toxins in the cecal contents of that two hamsters that came down with the disease after *sinRR’* mutant challenge. Since nearly 80% of the animals survived the R20291::*sinRR’* challenge, we conclude that members of the SinRR’ regulon are needed for *C*. *difficile* successful pathogenesis.

## Discussion

This study aims to decipher the role of the SinRR’ regulators in *C*. *difficile* physiology. In *C*. *difficile*, there has been no data explaining their function, except for a few expression analyses, where mutations in *sigH*, *tcdR*, *codY*, *spo0A*, *opp*, *app* were found to affect the expression of the *sin* locus [13, 14, 21, 22, 60]. Initial clues about the role of SinR and SinR’ in sporulation came from the work performed by Saujet *et al*. where they showed increased expression of *sinR* in the asporogenous *sigH* mutant, suggesting it to be a negative regulator of sporulation as in the case of *B*. *subtilis* [22]. However, *sinR* was found to be up-regulated in the hyper-sporulating oligopeptide transporter *opp-app* mutant and was down-regulated in the hypo-sporulation *tcdR* mutant [13, 60]. These later studies suggested the positive influence of SinR on sporulation. In this work, we mutated the *sin* locus in two different *C*. *difficile* strains and conclusively showed that unlike *B*. *subtilis* SinR, which inhibits sporulation, *C*. *difficile* SinR has a positive effect on sporulation.

Transcriptome analysis of *sinRR’* mutants revealed that in addition to sporulation, genes involved in motility, transport, stress response, cell wall biogenesis, and various metabolic pathways were also affected. It is worth noting that *cynT*, the gene adjacent to *sin* locus (Fig 1B), is one among the many metabolic genes that were found to be down-regulated in the *sinRR’* mutants (S4 and S6 Tables). The analysis also revealed that the *sin* locus mutations could affect the transcription of many important regulators, including *codY*, *sigD*, *spo0A*, and *tcdR*. This observation compelled us to hypothesize that SinRR’ might be indirectly influencing transcription of many of these genes by controlling their regulators. For example, changing in the transcription of *codY*, a global regulator can affect the gene regulatory circuits of various pathways.

**Fig 11:**
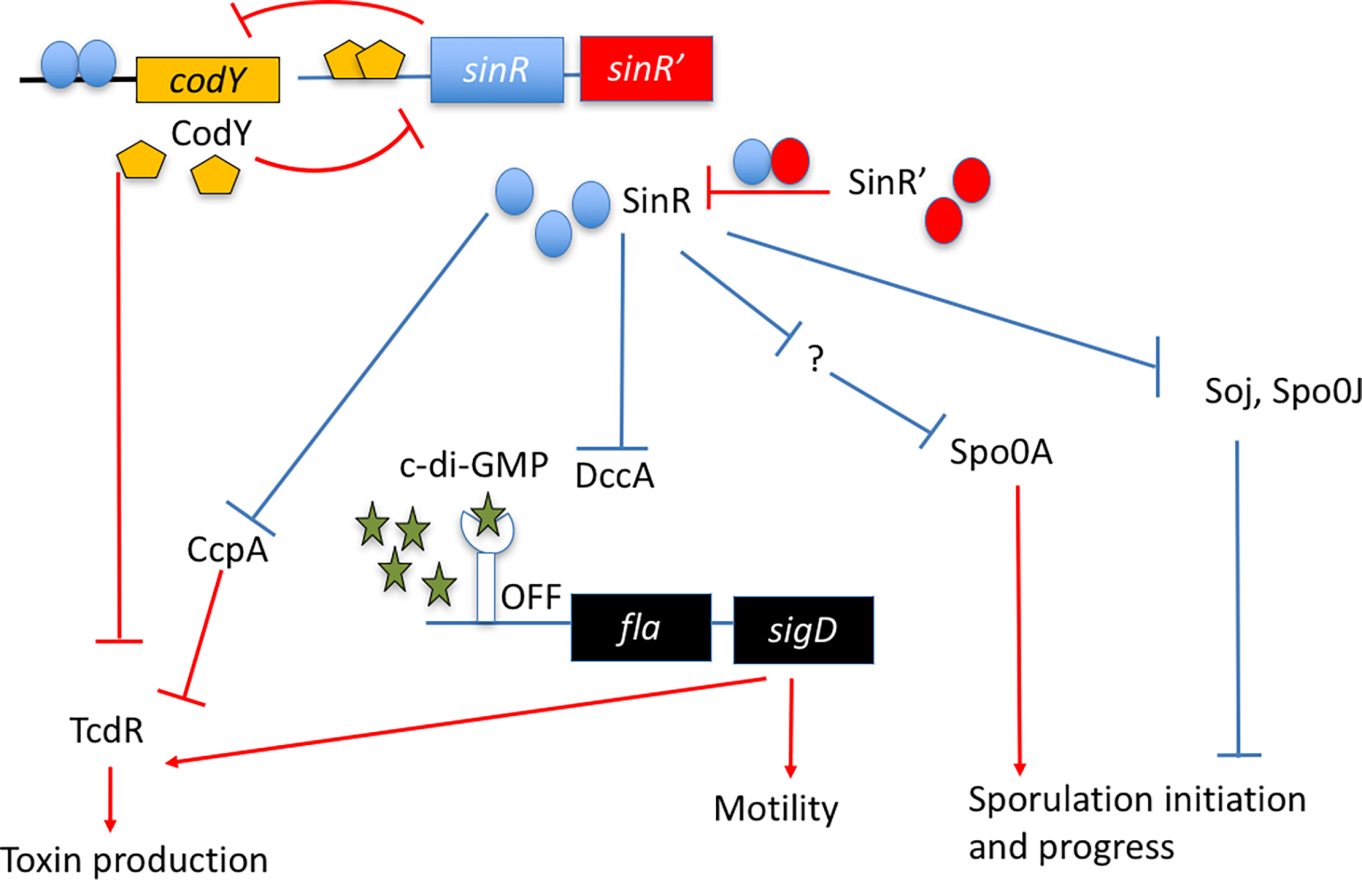
Schematic diagram showing *sin* locus regulation of genes involved in toxin production, sporulation, and motility in *C. difficile*. Known genetic interactions are marked in red and the predicted interactions are marked in blue.

CodY is known to be a sensor of the metabolic state of the cell. During the exponential growth phase, when the nutrients are abundant, CodY binds to branched-chain amino acids (BCAAs), and GTP and acts primarily as a repressor of various alternative metabolic pathways [17, 18]. When nutrients become limited in the cell, CodY is no longer bound by the cofactors and the transcriptional repression by CodY is alleviated on its targets. In *C*. *difficile*, CodY controls toxin production and sporulation in addition to metabolic pathways [14, 17, 18]. The transcription of *codY* was found to be up-regulated in the R20291::*sinRR’* (S5 Table), (Fig 11). This observation of increased *codY* transcription in the asporogenic *sinRR’ C*. *difficile* mutant is consistent with the recent findings that a *C*. *difficile codY* mutant hyper- sporulates [14]. To test whether increased CodY activity in the mutant is the reason for its lower toxin production and sporulation, we tried to isolate a *sinRR’*-*codY* double mutant and were unsuccessful even after several attempts. However, our EMSA experiments with purified SinR and *codY* upstream DNA showed that the SinR could specifically bind to this region, possibly to repress its transcription. We have also shown that purified CodY, in turn, can bind with the upstream region of *sin* locus upstream region to control its expression. Since the *sin* locus codes for both *sinR* and its antagonist *sinR’*, SinR repression on *codY* would be moderate when compared to CodY’s repression on the *sin* locus. Also, when the cells enter the stationary phase, CodY repression on the *sin* locus may be alleviated in the absence of its co-substrates and will result in the *sin locus* expression, which we found to be essential for sporulation initiation. We performed dot blot analysis with cytosolic proteins of R20291 and R20291::*sinRR’* and determined that CodY in R20291::*sinRR’* was only moderately higher than R20291 (S12 Fig). This could be due to the cell to cell variation in gene expression within the test population. For example, only 18% of the R20291 population enters sporulation in the growth conditions we tested. In *C*. *difficile*, only cells with low or inactive CodY enter sporulation. If we consider sporulation as an indirect measure for inactive CodY in a bacterial cell, we can say that the CodY production or activity was affected only in a fraction of cells in the parent population. To overcome this issue, we compared the CodY content in R20291::*sinR’* cells (which produce more SinR) with R20291::*sinRR’*. It is worth to note that nearly 50% of R20291::*sinR’* culture enters sporulation. Nearly two-fold more CodY could be detected in R20291::*sinRR’* cells when compared to R20291::*sinR’* cells. Other than modulating CodY content in *C*. *difficile*, SinR could also affect the CodY activity indirectly by affecting the concentrations of CodY substrates (BCAA and GTP). The transcriptome analysis indeed showed numerous metabolic genes to be affected in the *sinRR’* mutant.

In the JIR8094::*sinRR’* mutant, *codY* was not among the differentially regulated genes. However, in this strain *ccpA* was up-regulated nearly 13.5-fold (S7 Table). Similar to CodY, CcpA also represses toxin gene expression in *C*. *difficile* [15, 16]. Thus lower toxin production in JIR8094::*sinRR’* could be due to the higher CcpA activity in this mutant (Fig 11). We are currently testing whether *ccpA* is directly regulated by SinRR’. We are also setting up experiments to check whether increased CcpA has any role in controlling *codY* expression in the JIR8094::*sinRR’* strain.

SigD is one other regulator whose expression was found to be affected in the *sinRR’* mutants. In *C*. *difficile*, the *sigD* expression is repressed by elevated levels of c-di-GMP [50]. The enzyme, diguanylate cyclase coded by *dccA* synthesize c-di-GMP from GTP. In this study, we have shown the expression of *dccA* is up-regulated in *sinRR’* mutant (S5 Table and Table 2) and the observation of three-fold higher intracellular concentration of c-di-GMP in the *sinRR’* mutant, corroborated the transcriptome data. These results suggest that SinR and SinR’ regulates motility and toxin production indirectly by regulating the c-di-GMP production. Another scenario that can result in higher intracellular c-di-GMP concentration is when c-di-GMP degrading phosphodiesterases are reduced within the cell. In *C*. *difficile*, *pdcA* codes for a c-di-GMP phosphodiesterases, and it was recently identified to be repressed by CodY [61]. RNA-Seq analysis did not identify *pdcA* as one among the differentially regulated genes in R20291::*sinRR’* strain. However, it was under-expressed nearly 4-fold in *JIR8094*::*sinRR’* (S6 Table) mutant. Increased CodY activity in the *sinRR’* mutant could indirectly result in increased c-di-GMP concentration, which in turn can suppress toxin production and motility.

In *B*. *subtilis*, the SinR’s repressor’s activity on its target genes is inhibited by SinI, which is coded in the same operon (Fig 1A). In *B*. *subtilis* the polycistronic *sinRI* transcripts are produced from two upstream promoters. The monocistronic *sinR* transcripts are driven from a promoter located within the coding region of *sinI*. Regulating the transcription rate of *sinRI* and *sinR* helps *B*. *subtilis* to control its SinR and SinI content. Our RT-PCR and QRT-PCR analysis detected *sinRR’* transcripts in *C*. *difficile*. We have also shown that disrupting *sinR* by insertion mutagenesis affects both *sinR* and *sinR’* transcription. These results suggest that *sinRR’* is transcribe as a bicistronic message. However, there is a possibility that *sinR’* may have an independent promoter within *sinR* coding sequence as in *B*. *subtilis*. Our QRT-PCR analysis repeatedly detected lower levels of *sinR’*, *sinRR’* transcripts than the *sinR* transcripts. Western blot analysis also revealed lower levels of SinR’ than the SinR in growth conditions tested (Fig 2B-lane 2and S9C Fig). There is a possibility that mRNA degradation from the 3’ end can result in lower levels of *sinR’* transcripts, which in turn can result in lower levels of SinR’ than SinR. We did not detect any secondary structures upstream of *sinR’* that can influence its translation rate or translation initiation. We, however, noted that the RBS of *sinR’* are just two nucleotides away from the *sinR* stop codon. Ribosome complex occupying the *sinR* stop codon can prevent the assembly of new ribosome complex at the *sinR’* RBS to initiate translation. Since SinR’ has a DNA binding domain, it is also possible that SinR’ may work as direct regulator independently from SinR and may have its own targets for regulation. In such case, SinR’ may not always be available to inhibit SinR function. A transcriptome analysis of *sinR’* mutant and its comparison with *sinRR’* transcriptome may help us to identify direct targets of SinR’.

In *B*. *subtilis*, other than SinI, SinR also interacts with SlrR and SlrA to regulate genes involved in matrix formation (the *eps* and *tap-sipW-tas* operon), autolysis (*lytABC*) and motility (hag, encoding flagellin) [29, 31]. In *B*. *subtilis*, the SlrR is a DNA binding protein, and it is homologous to SinR. Conversely, SlrA is a small protein devoid of any DNA binding domains and is homologous to SinI [38, 39]. While SlrR can form heterodimers with SinR to repress *lytABC* and *hag* expression, it can also inhibit SinR’s repression activity on *eps* and *tap-sipW-tas* operons [38, 39, 62] which are needed for biofilm formation. The *C*. *difficile sin* locus codes for two DNA binding proteins SinR and SinR’ and their interactions resembles the interaction between *B*. *subtilis* SinR-SlrR. Similar to *B*. *subtilis* SlrR, *C*. *difficile* SinR’ carries a DNA binding domain and it will be interesting to analyze whether SinR-SinR’ complexes together are needed for the repression of any genes. It is important to note that the autolysis phenotype of *sinRR’* mutant was complemented only when both SinR and SinR’ were expressed (Fig 2C). This suggests that like *B*. *subtilis* SinR-SlrR complex, SinR-SinR’ complex together repress autolysis in *C*. *difficile*. No *lytABC* homologs could be identified in *C*. *difficile* genome, and the precise reason for autolysis in *sinRR’* mutant is not clear yet. However, the RNA-Seq data revealed that nearly 6% of the differentially expressed genes in *sinRR’* mutant plays a role in cell wall synthesis or assembly. This highlights that SinR and SinR’ play an important regulatory role in this pathway.

Among the phenotypes tested, asporogenesis of the *sinRR’* mutant was the only one we could not complement. Even the expression of *spo0A* failed to initiate sporulation in this mutant. Transcripts of Spo0A~P activated *sigE* and *sigF* did not show any increase when *spo0A* was expressed in the *sinRR’* mutant, suggesting the Spo0A remain unphosphorylated and inactive. We are currently performing additional experiments to test this hypothesis.

Another regulatory checkpoint for sporulation initiation is chromosomal DNA replication and segregation. This is achieved through the action of Soj and Spo0J in *B*. *subtilis*, where they repress sporulation until chromosomal segregation has occurred. They block the *spo0A* dependent transcription in *B*. *subtilis* [63]. The *spo0J* and *soj* homologs in *C*. *difficile* are CD630_3671 and CD630_3672, respectively in an operon, which also carries CD630_3673, an additional Spo0J-like orthologue. In both JIR8094::*sinRR’* and R20291::*sinRR’*, all three genes were up-regulated ~3 fold. Hence, the inactivation of Spo0A could result partly because of the up-regulation of the *soj* operon in the *sinRR’* mutants (Fig 11). But the function of *soj* and *spo0J* in *C*. *difficile* should be determined before we can speculate their roles in asporogenesis of *sinRR’* mutants.

BLAST search revealed that SinRR’ to be unique to *C*. *difficile* and its close relative *Clostridium sordellii*. The *sin* locus is absent in other *Clostridia*. Even though sporulation-specific sigma factors appear to be conserved among *Clostridia*, recent studies have suggested that sporulation initiation and regulation of *C*. *difficile* to be distinct [64, 65]. Since the *sin* locus appears to play a significant role in sporulation initiation and regulation, it is reasonable to speculate its presence could be one of the reasons why the regulation of sporulation initiation is distinct in *C*. *difficile*.

In summary, our study supports earlier reports that in *C*. *difficile*, virulence, sporulation, metabolism and motility pathways are inter-connected [13–24]. While many regulators in this network are yet to be identified, here we present the evidence that SinRR’ play a central role in this regulatory network. SinR regulates multiple pathways by controlling other global regulators. Finding genes that are directly under SinR regulation may lead to the identification of new regulatory genes and gene products that are important for *C*. *difficile* pathogenesis.

## Materials and methods

### Ethics statement

All animal procedures were performed with prior approval from the KSU Institutional Animal Care and Use Committee (protocol #3657). Animals showing signs of disease were euthanized by CO2 asphyxia followed by thoracotomy as a secondary means of death, in accordance with Panel on Euthanasia of the American Veterinary Medical Association. Kansas State University is accredited by AAALAC International (Unit #000667) and files an Assurance Statement with the NIH Office of Laboratory Animal Welfare (OLAW). KSU Animal Welfare Assurance Number is D16-00369 (A3609-01), and USDA Certificate Number is 48-R-0001. Kansas State University utilizes the United States Government Principles for the utilization and care of vertebrate animals used in testing, research and training guidelines for appropriate animal use in a research and teaching setting.

### Bacterial strains and growth conditions

Bacterial strains and plasmids used in this study are listed in S1 Table and cloning strategies used are listed in S1 Text. *Clostridium difficile* strains were grown anaerobically (10% H_2_, 10% C0_2_ and 80% N_2_) in TY (Tryptose and Yeast extract) agar or broth as described previously [60, 66]. Erythromycin (Erm; 2.5 μg ml^-1^), Lincomycin (Linc 20ug/ml), Cefoxitin (Cef; 25 μg/ml), thiamphenicol (Thio; 15 μg ml^-1^) were added to culture medium whenever necessary. Sporulation was induced in respective *C*. *difficile* strains by growing them in 70:30 sporulation medium (63 g Bacto-Peptone, 3.5 g Protease-Peptone, 11.1 g BHI, 1.5 g Yeast-Extract, 1.06 g Tris base, 0.7 g NH_4_SO_4_, 15 g agar per liter) [67]. *Escherichia coli* strain S17-1 [68] was used for conjugation and cultured aerobically in Luria-Bertani (LB) broth and supplemented with chloramphenicol (25μg ml^-1^) or ampicillin (100μg ml^-1^) as indicated.

### Construction of *C*. *difficile* mutant strains

ClosTron gene knockout system [69] was used to construct *sinRR’* and *sinR’* mutants. For *sinRR’* disruption, the group II intron insertion site between nucleotides 141 and 142 in *sinR* gene in the antisense orientation was selected using the Perutka algorithm, a Web-based design tool available at http://www.Clostron.com. For *sinR’* mutant construction, the group II intron insertion site between nucleotides 129 and 130 in the sense direction was selected. The designed retargeted intron was cloned into pMTL007-CE5 as described previously [59, 70]. The resulting plasmids pMTL007-CE5::Cdi-*sin*R-141s or pMTL007-CE5::Cdi-*sin*R’-129s was transferred into *C*. *difficile* cells by conjugation as described earlier [59, 70]. The potential Ll.ltrB insertions within the target genes in the *C*. *difficile* chromosome was conferred by the selection of erythromycin or lincomycin resistant transconjugants in 5 μg ml^-1^erythromycin or 20 μg ml^-1^ lincomycin plates. PCR using gene-specific primers (S2 Table) in combination with the EBS-U universal and ERM primers was performed to identify putative *C*. *difficile* mutants.

### General DNA techniques

DNeasy Blood and Tissue Kit (Qiagen) was used to extract chromosomal DNA from the *C*. *difficile* cultures. Primers used throughout the study are listed in S2 Table and S3 Table. Geneclean Kit (mpbio) was used to gel extract the PCR products, and QIAprep Spin Miniprep Kit (Qiagen) was used to extract plasmid DNA. Standard procedures were used to perform routine cloning.

### Sporulation efficiency assays

Sporulation assays were performed in 70:30 sporulation medium as described previously [60]. *C*. *difficile* strains were grown on 70:30 sporulation agar. After 30 h of growth, cells were scraped from the plates and suspended in 70:30 sporulation liquid medium to an OD_600_ of 1.0. Cells were immediately serially diluted and plated onto TY agar with 0.1% taurocholate to enumerate viable vegetative cells and spores. To determine the number of spores present, 500μl of the samples from each culture were mixed 1:1 with 95% ethanol and incubated for 1hour to kill all the vegetative cells. The ethanol-treated samples were then serially diluted, plated on TY agar with 0.1% taurocholate and incubated at 37^°^C for 24 to 48 hours to enumerate the number of spores. Dividing the number of spores by the total number of CFU and multiplying the value by 100 determined the percentage of ethanol-resistant spores. The results were based on a minimum of three biological replicates.

### Phase-contrast microscopy

*C*. *difficile* strains were grown in 70:30 medium as described above. At indicated time points, 1 ml of culture was removed from the anaerobic chamber, centrifuged at 17,000g for 1min and suspended in 30μl of sterile PBS. A thin layer of 0.7% agarose was applied to the surface of slide and 2μl of concentrated culture was placed on it. Phase contrast microscopy was performed using 100x oil immersion objective on OLYMPUS BX41 microscope. The PixeLINK camera was used to acquire the view of at least three fields for each strain.

### Transmission electron microscopy

All steps in sample preparation were performed at room temperature and solutions were prepared in 1X PBS (phosphate-buffered saline) unless indicated otherwise. For transmission electron microscopy, cells (10^10^) were fixed overnight in a solution of 2% glutaraldehyde and 2% paraformaldehyde. The cells were thoroughly rinsed with 1X PBS (5 minutes each) and post-fixed with 1% osmium tetroxide with constant rotation for 1–2 hours. The samples were then washed thrice with 1X PBS (5 minutes each), enblock stained with 2% Uranyl acetate in water for 1hr with light protection, and finally washed three times (5 min each) with distilled water. The cells were further dehydrated in a graded 50% -100% acetone series (vol/vol) for 5 minutes and infiltrated in graded EMBED 812/Araldite resin (Electron Microscopy Sciences) at RT with constant rotation. Thin sections of polymerized resin were placed on copper grids and stained with 2% alcoholic uranyl acetate and Reynolds' lead citrate respectively. Sections were examined with a transmission electron microscope (Philips CM100) and regions containing the cross-section of the cells were photographed at 80 kV for image analysis.

To visualize the flagella, whole bacterial cells harvested from overnight cultures were processed as above and were negatively stained with 2% uranyl acetate before transmission electron microscopy analysis.

### RNA-Seq analysis

We isolated total RNA from three biological replicates of each strain belonging to early-stationary phase (12 hours after inoculation) and quality was checked using Agilent 2100 Bioanalyzer. The RNA-Seq was performed as previously described [60]. Briefly, we depleted the rRNA content in the selected samples using Epicenter Bacterial Ribo-Zero kit. Strand-specific single end cDNA libraries were prepared using Truseq Small Stranded Total RNA sample prep kit Illumina as per the manufacturers’ instructions. Illumina HiSeq2000 sequencer (multiplexing three samples per lane) was used to sequence libraries. Sequences were cleaned with AlienTrimmer [71] of adapter sequences. Only high-quality sequences with a minimum of 30 nucleotides in length were considered for further analysis. Cleaned genes were aligned to reference genomes (FN545816.1 and AM180355.1) using Bowtie (version 1.0.1) [25, 60, 72]. DESeq2 version 1.8.3 was used to perform normalization and differential analysis. Genes were considered differentially expressed if the fold change was ≥ log_2_ 1.5 and their adjusted *p*-value was ≤0.05.

### Cloning, expression, and purification of SinR-6His, SinR’-6His, and CodY-6His proteins in *E*. *coli*

SinR, SinR’ and CodY proteins were overexpressed in Rosetta *E*. *coli* DE3 cells using pET16B expression system. The ORFs for cloning were PCR amplified from JIR8094 chromosome using gene-specific primers (listed in S2 Table), and the amplified gene fragments were then digested with *Xho1* and *BamH1* to clone into pET16B digested with the same enzymes. The resulting plasmids were then transformed into *E*.*coli* Rosetta DE3 (Novagen) competent cells to obtain recombinant strains. To overexpress SinR-6His, and SinR’-6His, the *E*. *coli* recombinant strains were grown at 37^°^C in LB medium containing chloramphenicol (25μg ml^-1^) and ampicillin (100ug ml^-1^). Protein expression was achieved by inducing with 1mM IPTG at 17^°^C overnight. Cells were harvested by centrifugation, and the 6His-tagged proteins were purified by affinity chromatography on Ni^++^ agarose (Sigma-Aldrich) beads following the manufacturer’s recommendations.

### Antibody production

The anti-SinR used in this study was raised against SinR-His_6_ in rabbits by Lampire Biologicals (Everett, PA). The anti-SinR’ was raised against SinR’-His_6_ in mice by Lampire Biologicals (Everett, PA).

### Western blot analysis

*C*. *difficile* cells for western blot analysis were harvested and washed in 1x PBS solution before suspending in sample buffer (Tris 80mM; SDS 2%; and Glycerol 10%) for sonication. Whole cell extracts were then heated at 100^°^C for 7 min and centrifuged at 17,000 g for 1 min, and the proteins were separated by SDS-PAGE and electro-blotted onto PVDF membrane. Immobilized proteins in the membranes were then probed with specific antibodies at a dilution of 1:10,000. The blot was subsequently probed with HRP-conjugated secondary antibodies at a dilution of 1:10000. Immuno-detection of proteins was performed with ECL Kit (Thermo Scientific) following the manufacturer’s recommendations and were developed using Typhoon 9100 scanner.

### Toxin ELISA

Cytosolic toxins from 16h old *C*. *difficile* cultures grown in TY medium were measured as described previously [70, 73]. In brief, one ml of *C*. *difficile* cultures were harvested and suspended in 200 μl of sterile PBS, sonicated and centrifuged to harvest the cytosolic protein. One hundred μg of cytosolic proteins was used to measure the relative toxin levels using *C*. *difficile* premier Toxin A &B ELISA kit from Meridian Diagnostics Inc. (Cincinnati, OH).

### Motility assay

*C*. *difficile* cultures were grown until mid-exponential phase at 37^°^C. After adjusting their OD@600 to 0.5, 3μl of each strain was inoculated by stabbing or spotting into BHI medium with 0.3% w/v agar in tubes and plates respectively. After incubation at 37^°^C, the motility was quantified by measuring the radius of the cultures at different time points. Motility assay was performed in 4 replicates and independently repeated at least three times.

### SinR-6His; SinI-GST pull-down experiment

To express SinR’-GST protein we cloned the *sinR’* gene in the pGST-parallel2 expression system [74]. First, the *sinR’* gene was PCR amplified using primers ORG619 and ORG620 (S2 Table) and R20291 chromosomal DNA as a template. The PCR fragments were then cloned in between *NcoI* and *SalI* sites of the pGST-parallel2 vector. The resulting plasmid was then transformed into *E*.*coli* Rosetta DE3 competent cells to obtain recombinant strain. To overexpress SinR’-GST, *E*. *coli* recombinant strains were grown at 37^°^C in LB medium containing chloramphenicol (25μg ml^-1^). Protein expression was achieved by inducing with 1mM IPTG at 17^°^C overnight with mild agitation. To perform the pull-down experiment, 200 μgs of whole cell lysate proteins from the *E*. *coli* cells expressing SinR’-GST was mixed with ~20 μgs of purified SinR-6His protein and incubated at 4^°^C for 1hr. The mixture was then passed through the Ni^++^ affinity column (Sigma-Aldrich) to trap and elute SinR-6His protein. Whole lysates from *E*. *coli* cells expressing GST alone was also mixed with purified SinR-6His protein, and this control mixture was processed in the same way as the test sample. The elutes from Ni++ columns were then separated by SDS-PAGE and were electro blotted onto PVDF membrane. Membranes with immobilized proteins were then probed with either Anti-6His antibodies at 1:10,000 dilution or with anti-GST antibodies at the dilution of 1:5000. Immunodetection of proteins was performed with Pierce ECL 2 Western blotting Substrate Kit (Thermo Scientific) and the Typhoon 9100 scanner.

### Electrophoretic mobility shift assay (EMSAs)

SinR and SinR’ binding was performed with radioactively labeled DNA probes. The *codY* upstream and the *gluD* upstream regions were amplified using primer pairs ORG629- ORG630 and ORG72-ORG73, respectively and the products were cloned into a pGEMT cloning vector. The region was then excised from the plasmid construct using *EcoRI* and was radiolabeled using Klenow fragment of DNA polymerase I (NEB. labs) and [α- ^32^ P]dATP-6000 Ci/mmol (PerkinElmer Life Sciences). Binding experiments with radioactively labelled *codY* upstream DNA with SinR-6His or SinR’-6His was performed using reaction buffer containing 10 mM Tris–HCl (pH 8.0), 0.1 mM DTT, 150 mM KCl, 0.5mM EDTA, 0.1% Triton X-100 and 12.5% glycerol. For binding experiments containing both SinR and SinR’, proteins were mixed in the reaction buffer at a specified concentration and were incubated at room temperature for 30 minutes before adding the DNA probe. Reactions were loaded onto a 6% native polyacrylamide gel in 1XTBE (Tris/Borate/EDTA) and subjected to electrophoresis at 100 V for 45 minutes. Gels were then dried, and the autoradiography was performed with Molecular Dynamics Phosphor-Imager technology.

For the CodY binding experiments, the upstream region of the *sin* locus with the predicted CodY binding sequence (shown as underlined) 5’ TAGAAA ATTTTTTTAATTTTCAAAATATATTCTACATATCTAA was synthesized and was labeled with [γ- ^32^ P]dATP-6000 Ci/mmol (PerkinElmer Life Sciences) using T4 polynucleotide kinase. It was then annealed with the complementary oligo to generate double-stranded DNA probe. Known CodY binding sequence upstream of the *tcdR* gene was similarly synthesized (S2 Table) and used as a positive control. A non-specific double-stranded DNA was used as negative control (S2 Table). The DNA-protein binding reactions were carried out at room temperature for 30 min in 10μl volume containing 1x binding buffer [10mM Tris pH 7.5, 50mM KCl, 50μg BSA, 0.05% NP40, 10% Glycerol, 10 mM GTP and 2mM ILV (Isoleucine, Leucine and Valine), 100 μg/ml poly dI-dC and 800nM of DNA probe with varying concentration of purified CodY protein. DNA probe in reaction buffer was incubated for 10 min at RT before adding purified CodY-6His protein. The reaction was stopped by adding 5ul of gel loading buffer and electrophoresed at 100V for 1.5 h using 6% 1XTBE gel in 0.5X TBE buffer containing 10 mM ILV. Gels were then dried, and the autoradiography was performed with Molecular Dynamics Phosphor-Imager technology.

### Hamster model for *C*. *difficile* pathogenesis

Syrian golden hamsters (100–120 g) were used for *C*. *difficile* infection. Upon their arrival, fecal pellets were collected from all hamsters, homogenized in 1 ml saline, and examined for *C*. *difficile* by plating on CCFA-TA (Cycloserine Cefoxitin Fructose Agar- 0.1% Taurocholate) to ensure that the animals did not harbor indigenous *C*. *difficile*. After this initial screen, they were housed individually in sterile cages with *ad libitum* access to food and water for the duration of the study. Hamsters were first gavaged with 30 mg/kg clindamycin [59, 75]. *C*. *difficile* infection was initiated five days after clindamycin administration by gavage with vegetative cells. We used vegetative *C*. *difficile* cells because of the test strain R20291::*sinRR*’ is asporogenic and do not produce any spores. Bacterial inoculums were standardized and prepared immediately before challenge as described in our earlier study [59]. They were transported in independent 1.5 ml Eppendorf tubes to the vivarium using the Remel AnaeroPack system (one box for each strain) to maintain viability. Immediately before and after infecting the animal, a 10 μL sample of the inoculum was plated onto TY agar with cefoxitin to confirm the bacterial count and viability. There were five groups of animals, including the uninfected control group. Ten animals per group were used for the infection. Approximately, 2000 *C*. *difficile* vegetative cells of R20291 strain and R20291::*sinRR*’ were used for the animal challenge. In the uninfected control (group 5) only five animals were used, and they received only antibiotics and sterile PBS. Animals were monitored for signs of disease (lethargy, poor fur coat, sunken eyes, hunched posture, and wet tail) every four hours (six times per day) throughout the study period. Hamsters were scored from 1 to 5 for the signs mentioned above (1-normal and 5-severe). Fresh fecal pellets were collected daily from every animal to monitor *C*. *difficile* colonization until they began developing diarrheal symptoms. Hamsters showing signs of severe disease (a cumulative score of 12 or above) were euthanized by CO_2_ asphyxiation. Surviving hamsters were euthanized 15 days after *C*. *difficile* infection. Thoracotomy was performed as a secondary mean of death. The cecal contents from these hamsters were collected in 15ml Nalgene tubes, secured air tight and were transported to the lab using Remel AnaeroPack system. They were then immediately subjected to CFU enumeration. For CFU enumeration, the daily fecal samples or the cecal contents collected post-mortem were resuspended in 1X PBS, serially diluted and plated onto CCFA agar with 0.1% Taurocholate (CCFA-TA). The CFU were counted after 48 h of incubation. The survival data of the challenged animals were graphed as Kaplan-Meier survival analyses and compared for statistical significance using the log-rank test using GraphPad Prism 6 software (GraphPad Software, San Diego, CA).

## Supporting information

S1 Fig Construction and confirmation of the *sinR* mutant in *C*. *difficile* JIR8094 and R20291. (A) Schematic representation of ClostTron (group II intron)- mediated disruption of the *sinR* gene in *C*. *difficile*. (B) PCR verification of the intron insertion, conducted with intron-specific primer EBS universal [EBS(U)] with *sinR*—specific primers ORG-549 and ORG-550.
(TIF)

S2 Fig Evidence for the read-through transcription of *sin* locus in *C*. *difficile*. (A) RT-PCR results of *sinRR*’, *sinR* and *sinR’* using cDNA, RNA and genomic DNA prepared from *C*. *difficile* JIR8094 and JIR8094::*sinRR’*. (B) Schematic representation of gene structure in *sin* locus and the location of primer design site for each gene products respectively. (C) RT-PCR results of *sin* locus transcripts in JIR8094 and JIR8094::*sinRR’* strains collected at different time points. The representative results from three independent experiments are shown.
(TIF)

S3 Fig Characterizing *sinRR’* mutants. (A) Western blot analysis of parent and mutants using SinR and SinR’ specific antibodies. (B) Growth curve of parent and the mutant strains.
(TIF)

S4 Fig Autolysis assay. Triton X-100 induced autolysis of R20291::*sinRR’* at the stationary phase showing rapid lysis compared to the parent strain. Expression of *sinRR’* prevented autolysis in *sinRR’* mutant. The autolysis is expressed as percent initial absorbance at an optical density of 600nm. Error bars indicate ± standard deviation. The experiments were repeated at least three times independently (*, *p*≤0.05 by a two-tailed Student's *t*-test).
(TIF)

S5 Fig Analysis of sporulation in JIR8094::*sinRR’* mutant. (A) Phase contrast microscopy of JIR8094 and JIR8094::*sinRR’* cells. (B) JIR8904::*sinRR’* mutant was asporogenic as shown in the representative TEM images in comparison with the parent strain. Black arrows indicate mature spores in the parent strain. (C) Sporulation frequency of JIR8094 and JIR8094::*sinRR’* strains. The data shown are mean ± standard errors of three replicates. *** *p*< 0.0005 (by two-tailed student’s *t*-test). (D) Western blot analysis demonstrating lower Spo0A expression in the *sinRR’* mutant.
(TIF)

S6 Fig Motility analysis of *sinRR’* mutant. (A) Dot blot analysis of R20291, R20291::*sinRR’* proteins using FliC and GDH (internal control) specific antibody. (B) Swimming motility of the R20291 and R20291::*sinRR’* strain showing the non-motile phenotype of *sinRR’* mutant in BHIS with 0.3% agar.
(TIF)

S7 Fig Toxin production in JIR8094::*sinRR’* mutant. Toxin ELISA performed with cytosolic proteins harvested from JIR8094 and JIR8094::*sinRR’* mutant. The data shown are mean ± standard errors of three replicates. ** *p*< 0.005 (by two-tailed student’s t-test).
(TIF)

S8 Fig Quantification of intracellular c-di-GMP by HPLC. (A) The c-di-GMP peak in HPLC. (B) The standard curve was constructed by analyzing samples containing a predetermined amount of c-di-GMP and their respective peak area. (C) Analysis of intracellular nucleotide pools prepared from R20291 and R20291::*sinRR’* cells. Arrows indicate the peak corresponding to c-di-GMP.
(TIF)

S9 Fig Construction and characterization of the *sinR’* mutant in *C*. *difficile* R20291. **(A)** PCR verification of the intron insertion verified with intron-specific primer EBS universal [EBS(U)] with gene-specific primers ORG-553 and ORG-554. (B) Schematic representation of ClostTron (group II intron)- mediated disruption of the *sinR’* gene in *C*. *difficile* R20291. (C) Western blot analysis of R20291 and R20291::*sinR’* proteins using SinR and SinR’ specific antibodies. (D) Growth curve of parent R20291 and *sinR’* mutant in TY medium showing no autolysis of *sinR’* mutant.
(TIF)

S10 Fig Gel mobility shift assay reveals neither SinR nor SinR’ binds to *gluD* upstream (non-specific control DNA). (TIF)

S11 Fig Toxin ELISA to detect *C*. *difficile* toxins in cecal contents of infected hamsters. Cecal contents harvested upon post-mortem were analyzed using *C*. *difficile* premier Toxin A &B ELISA kit from Meridian Diagnostics Inc. (Cincinnati, OH), following manufacturer’s instruction. Negative control from the ELISA kit used along with the test samples. Each bar represents one animal.
(TIF)

S12 Fig Dot blot analysis of R20291, R20291::*sinRR’* and R20291::*sinR’* cytosolic proteins using CodY specific antibody. UK::*codY* mutant was used as a control.
(TIF)

S1 Table Bacterial strains and plasmids used in this study. (DOCX)

S2 Table Oligonucleotides used for PCR reactions. (DOCX)

S3 Table Oligonucleotides used for QRT-PCR reactions. (DOCX)

S4 Table Under-expressed genes in R20291::*sinRR’* compared to R20291. (XLSX)

S5 Table Over-expressed genes in R20291::*sinRR’* compared to R20291. (XLSX)

S6 Table Under-expressed genes in JIR8094::*sinRR’* compared to JIR8094. (XLSX)

S7 Table Over-expressed genes in JIR8094::*sinRR’* compared to JIR8094. (XLSX)

S8 Table QRT-PCR analysis of selected genes in *sinRR’* mutants. (DOCX)

S1 Text Plasmids construction. (DOCX)

S2 Text Supplemental methods. (DOCX)
