# Supplementary figures and images for: Pleiotropic roles of Clostridium difficile sin locus
Source: PLoS Pathog. 2018 Mar 12;14(3):e1006940. doi: 10.1371/journal.ppat.1006940 (PMC5864091; doi:10.1371/journal.ppat.1006940)

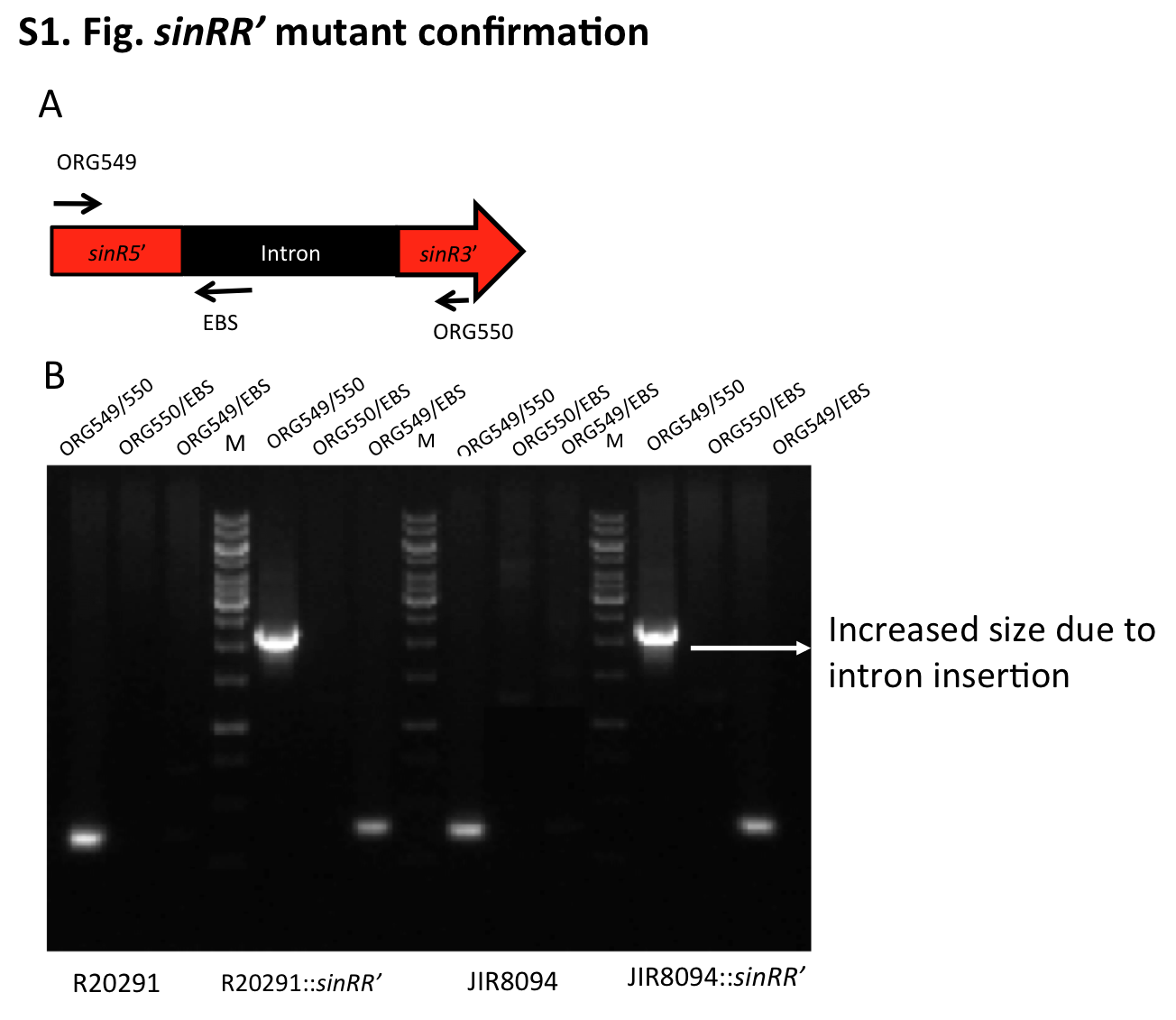

Supplement: S1 Fig — (A) Schematic representation of ClostTron (group II intron)- mediated disruption of the sinR gene in C. difficile. (B) PCR verification of the intron insertion, conducted with intron-specific primer EBS universal [EBS(U)] with sinR—specific primers ORG-549 and ORG-550. (TIF) [file ppat.1006940.s001.tif]

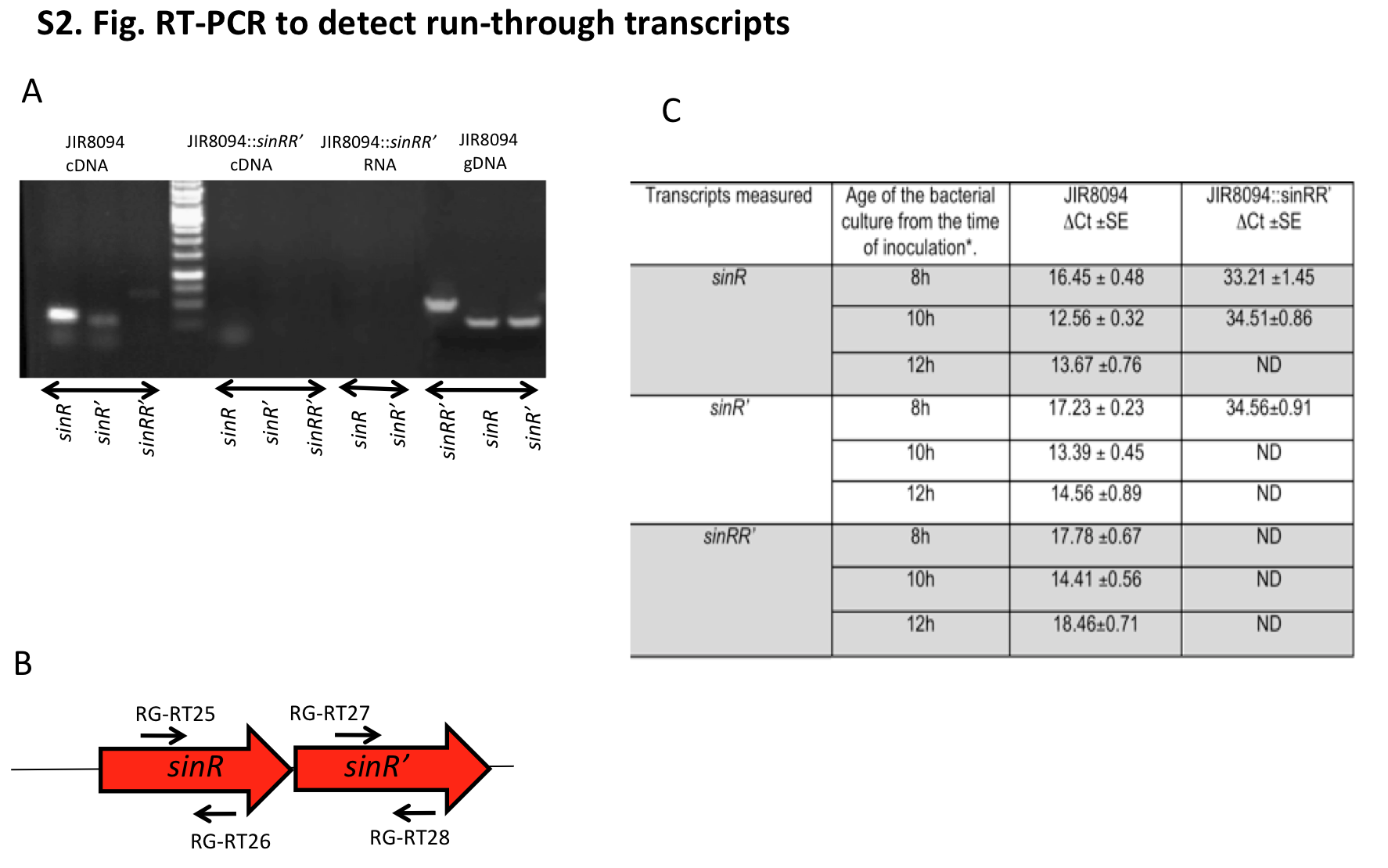

Supplement: S2 Fig — (A) RT-PCR results of sinRR’, sinR and sinR’ using cDNA, RNA and genomic DNA prepared from C. difficile JIR8094 and JIR8094::sinRR’. (B) Schematic representation of gene structure in sin locus and the location of primer design site for each gene products respectively. (C) RT-PCR results of sin locus transcripts in JIR8094 and JIR8094::sinRR’ strains collected at different time points. The representative results from three independent experiments are shown. (TIF) [file ppat.1006940.s002.tif]

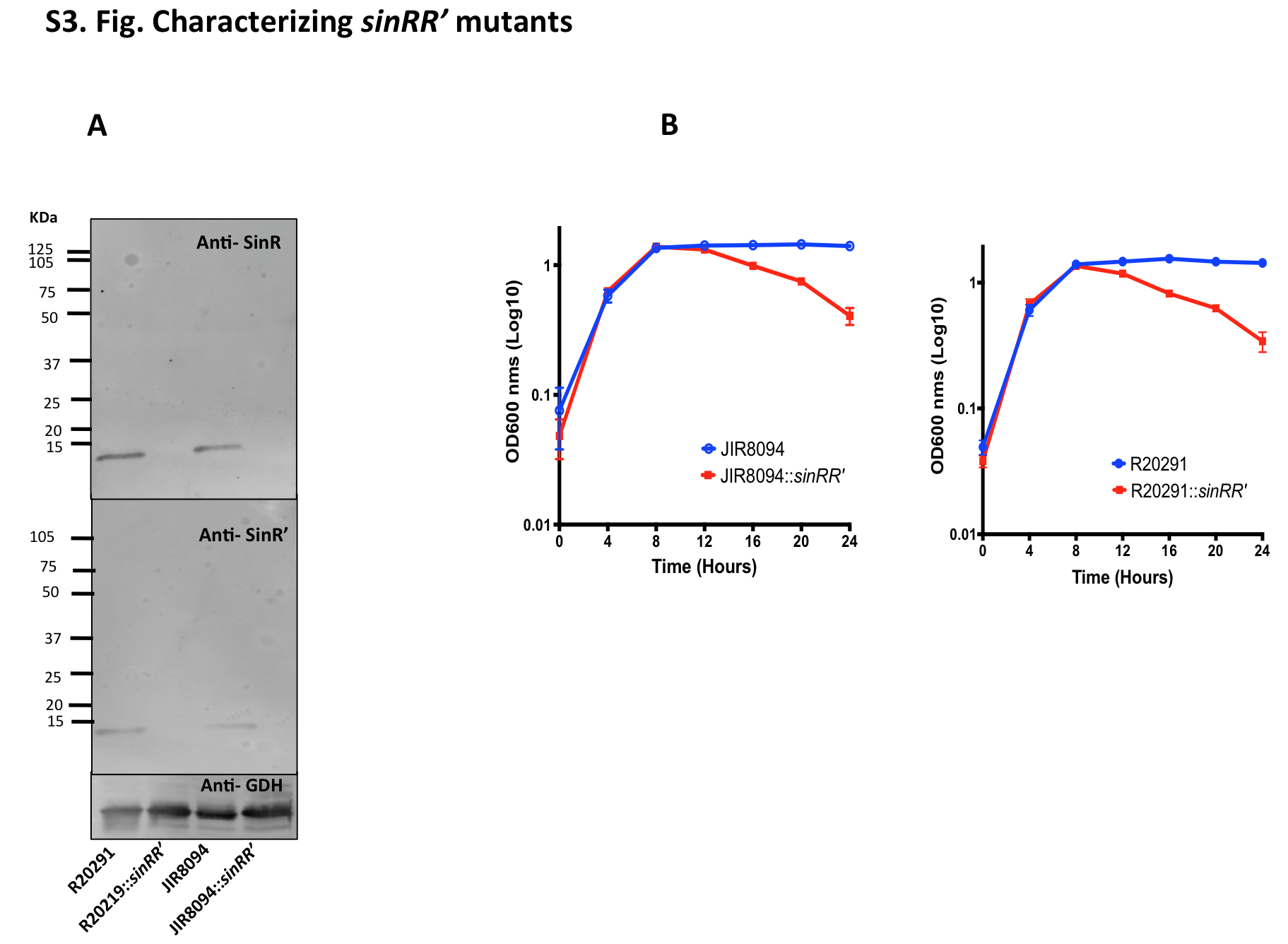

Supplement: S3 Fig — (A) Western blot analysis of parent and mutants using SinR and SinR’ specific antibodies. (B) Growth curve of parent and the mutant strains. (TIF) [file ppat.1006940.s003.tif]

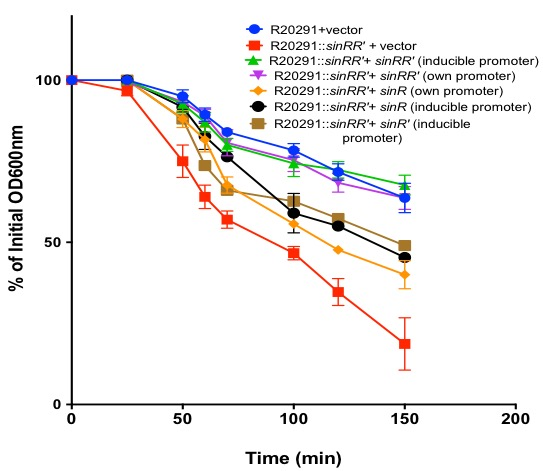

Supplement: S4 Fig — Triton X-100 induced autolysis of R20291::sinRR’ at the stationary phase showing rapid lysis compared to the parent strain. Expression of sinRR’ prevented autolysis in sinRR’ mutant. The autolysis is expressed as percent initial absorbance at an optical density of 600nm. Error bars indicate ± standard deviation. The experiments were repeated at least three times independently (*, p≤0.05 by a two-tailed Student's t-test). (TIF) [file ppat.1006940.s004.tif]

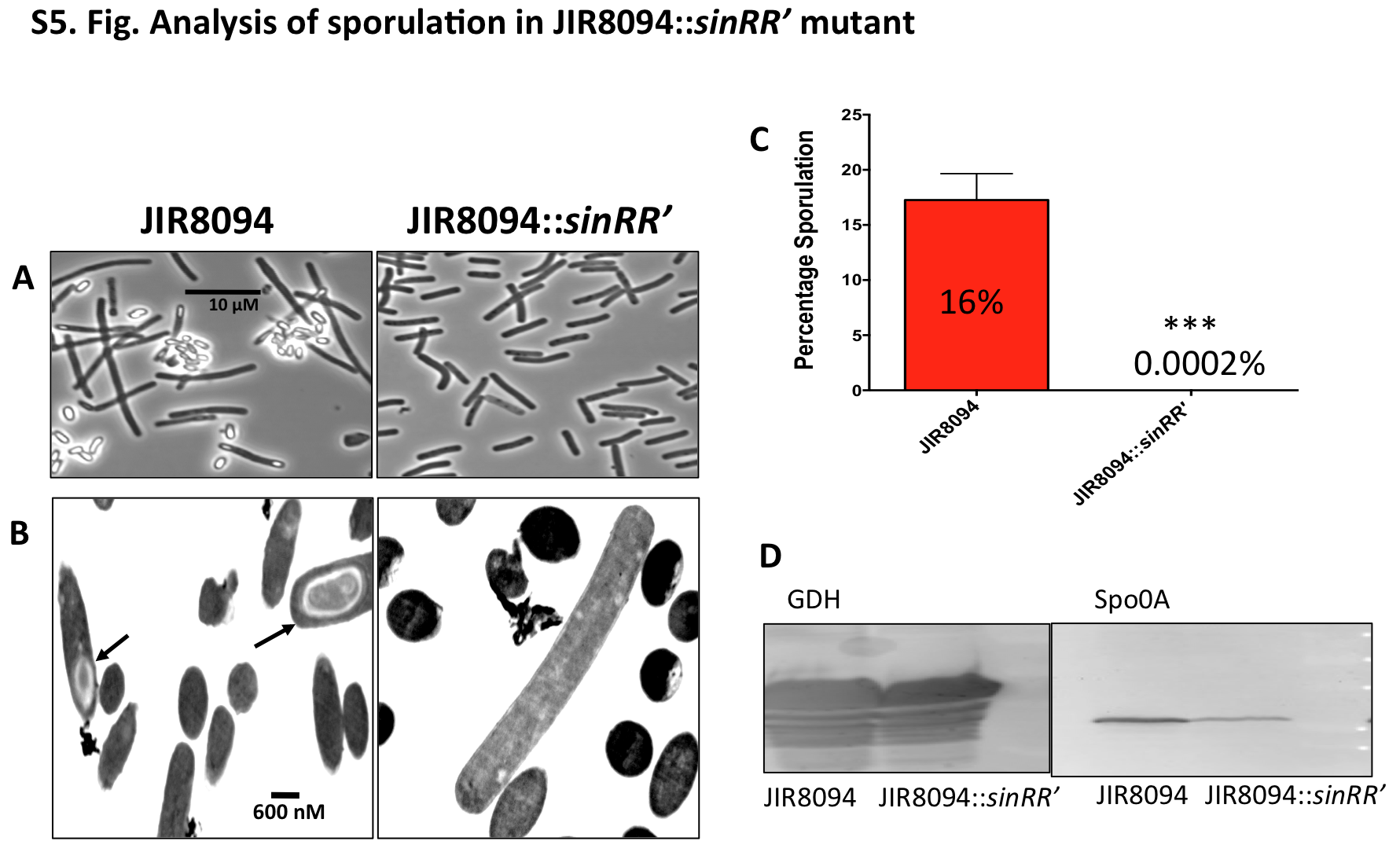

Supplement: S5 Fig — (A) Phase contrast microscopy of JIR8094 and JIR8094::sinRR’ cells. (B) JIR8904::sinRR’ mutant was asporogenic as shown in the representative TEM images in comparison with the parent strain. Black arrows indicate mature spores in the parent strain. (C) Sporulation frequency of JIR8094 and JIR8094::sinRR’ strains. The data shown are mean ± standard errors of three replicates. *** p< 0.0005 (by two-tailed student’s t-test). (D) Western blot analysis demonstrating lower Spo0A expression in the sinRR’ mutant. (TIF) [file ppat.1006940.s005.tif]

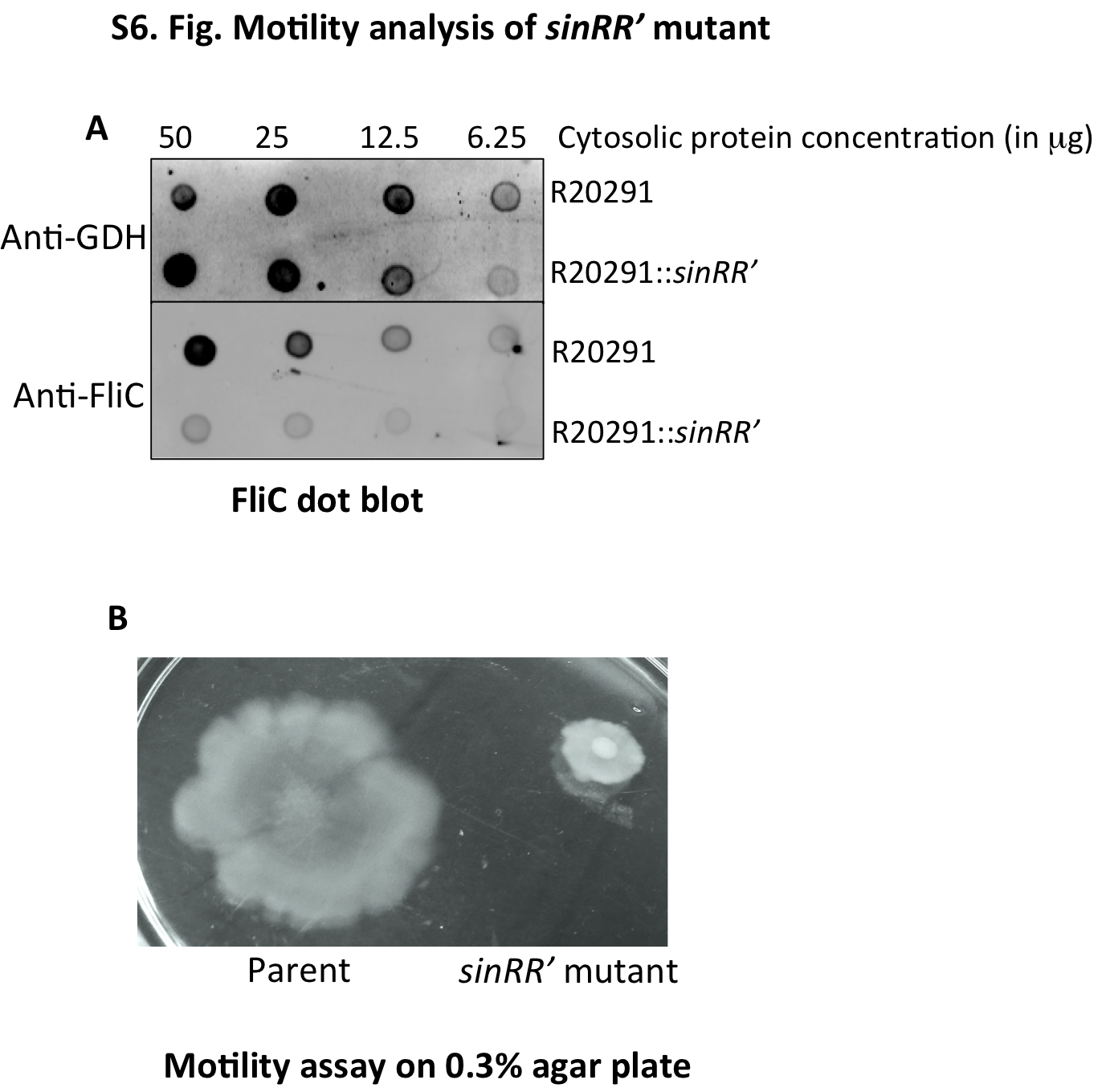

Supplement: S6 Fig — (A) Dot blot analysis of R20291, R20291::sinRR’ proteins using FliC and GDH (internal control) specific antibody. (B) Swimming motility of the R20291 and R20291::sinRR’ strain showing the non-motile phenotype of sinRR’ mutant in BHIS with 0.3% agar. (TIF) [file ppat.1006940.s006.tif]

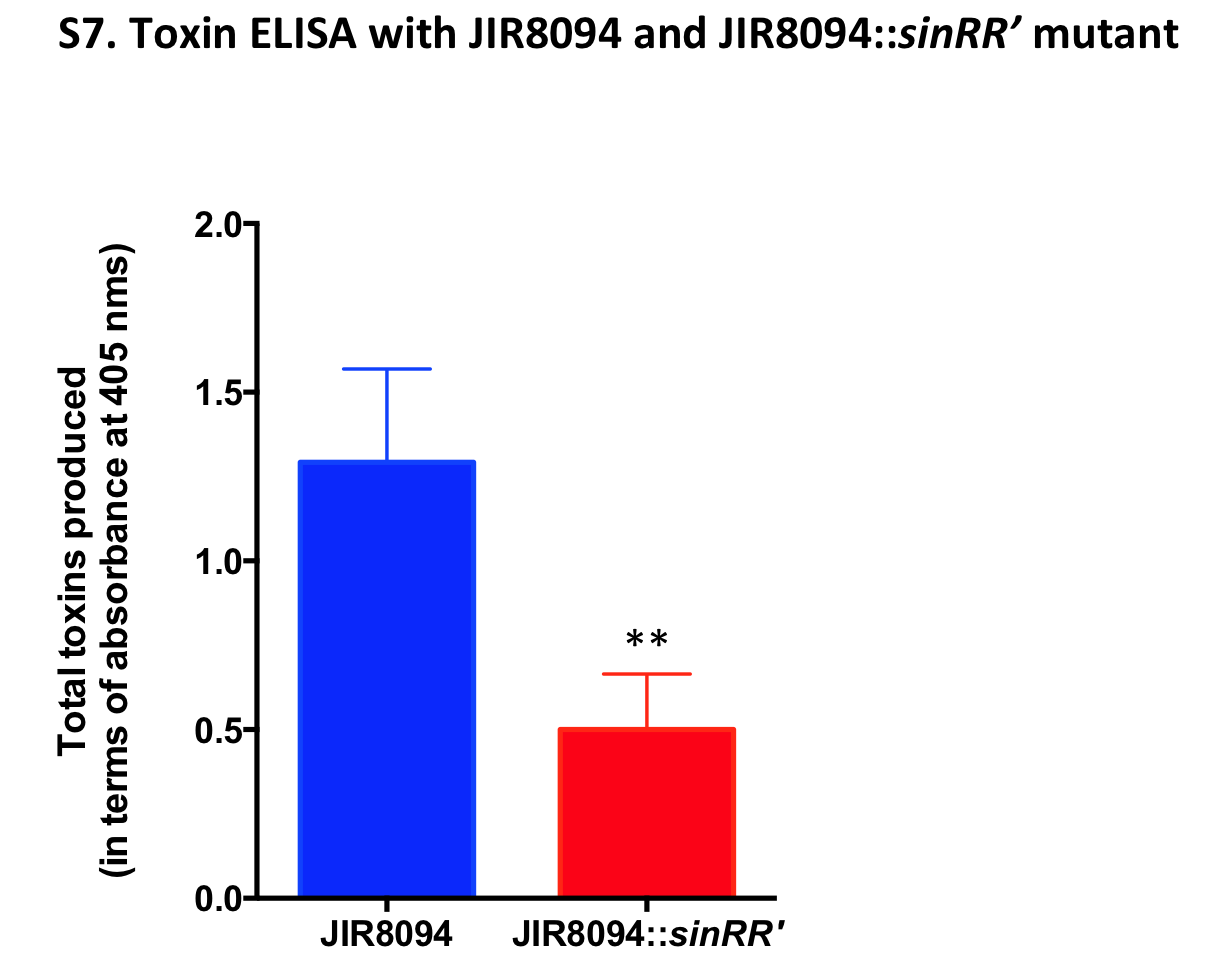

Supplement: S7 Fig — Toxin ELISA performed with cytosolic proteins harvested from JIR8094 and JIR8094::sinRR’ mutant. The data shown are mean ± standard errors of three replicates. ** p< 0.005 (by two-tailed student’s t-test). (TIF) [file ppat.1006940.s007.tif]

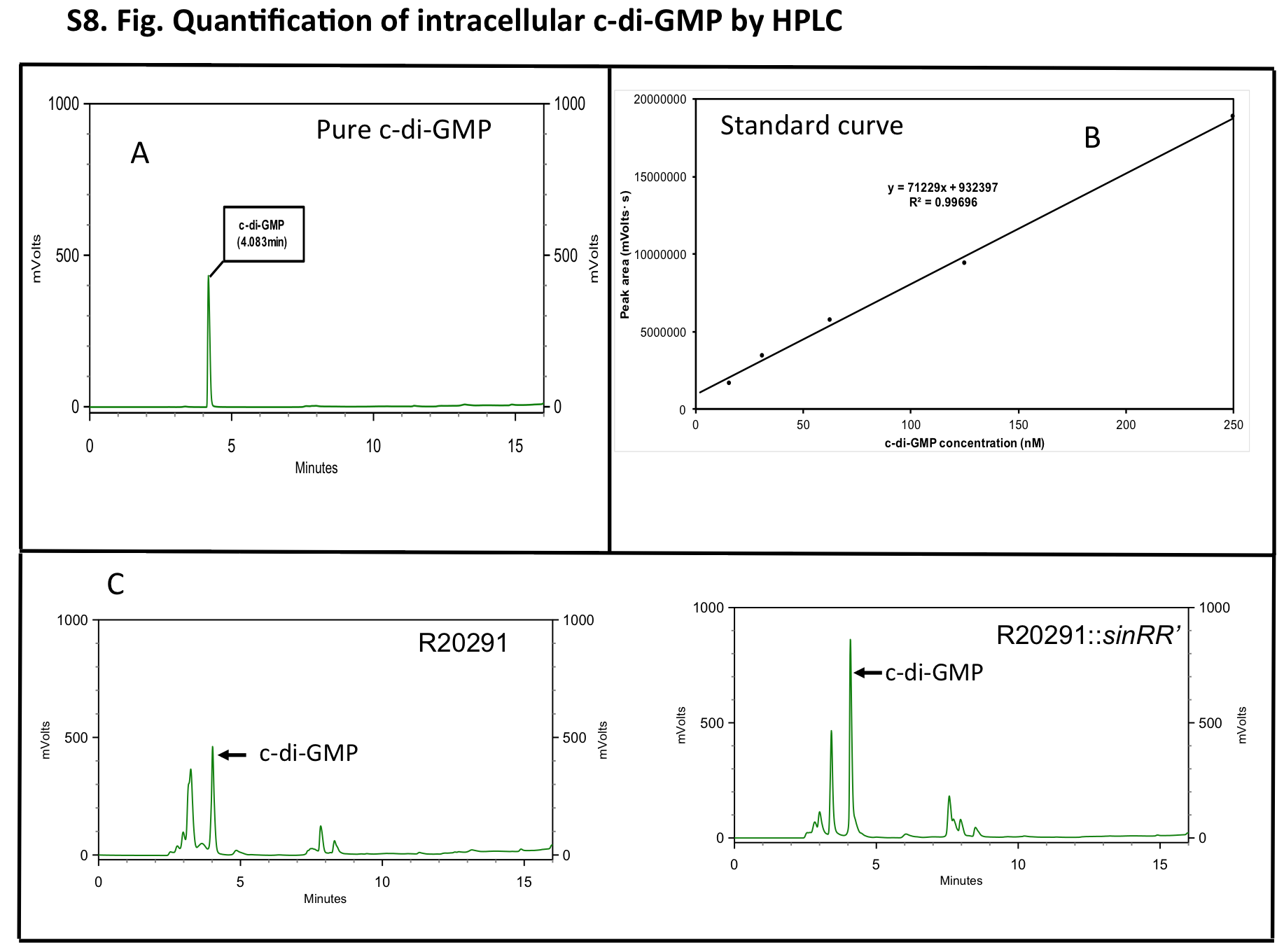

Supplement: S8 Fig — (A) The c-di-GMP peak in HPLC. (B) The standard curve was constructed by analyzing samples containing a predetermined amount of c-di-GMP and their respective peak area. (C) Analysis of intracellular nucleotide pools prepared from R20291 and R20291::sinRR’ cells. Arrows indicate the peak corresponding to c-di-GMP. (TIF) [file ppat.1006940.s008.tif]

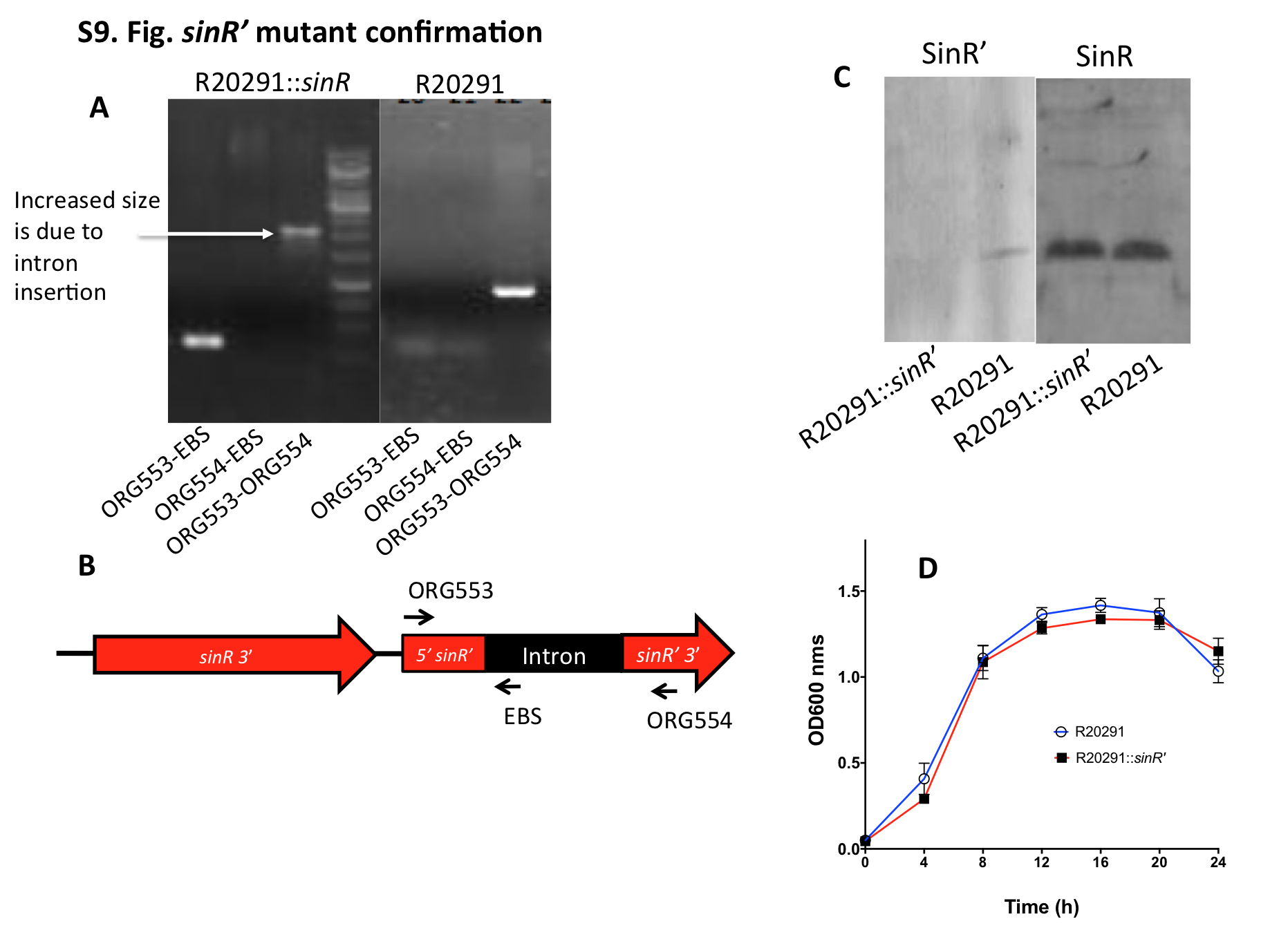

Supplement: S9 Fig — (A) PCR verification of the intron insertion verified with intron-specific primer EBS universal [EBS(U)] with gene-specific primers ORG-553 and ORG-554. (B) Schematic representation of ClostTron (group II intron)- mediated disruption of the sinR’ gene in C. difficile R20291. (C) Western blot analysis of R20291 and R20291::sinR’ proteins using SinR and SinR’ specific antibodies. (D) Growth curve of parent R20291 and sinR’ mutant in TY medium showing no autolysis of sinR’ mutant. (TIF) [file ppat.1006940.s009.tif]

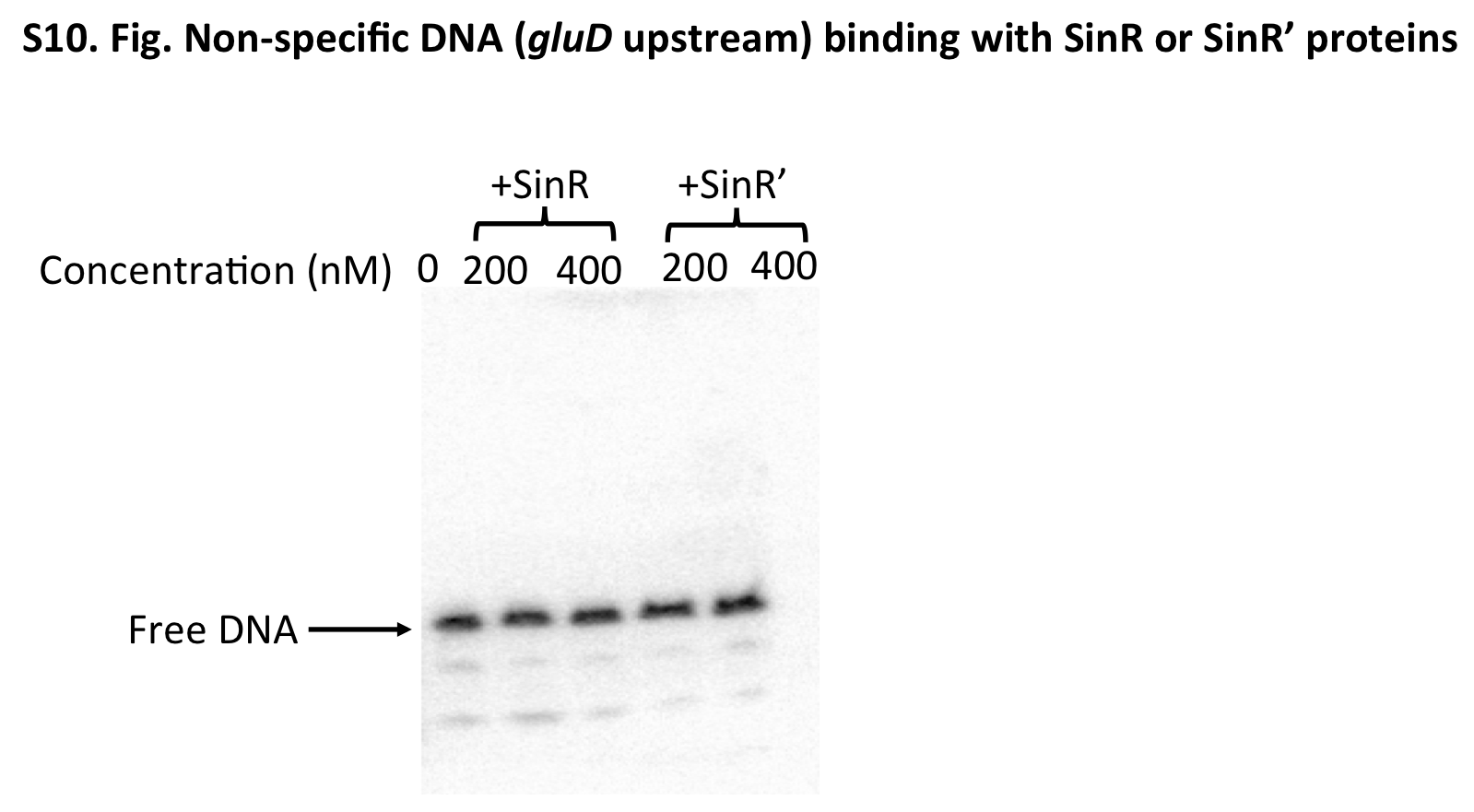

Supplement: S10 Fig — (TIF) [file ppat.1006940.s010.tif]

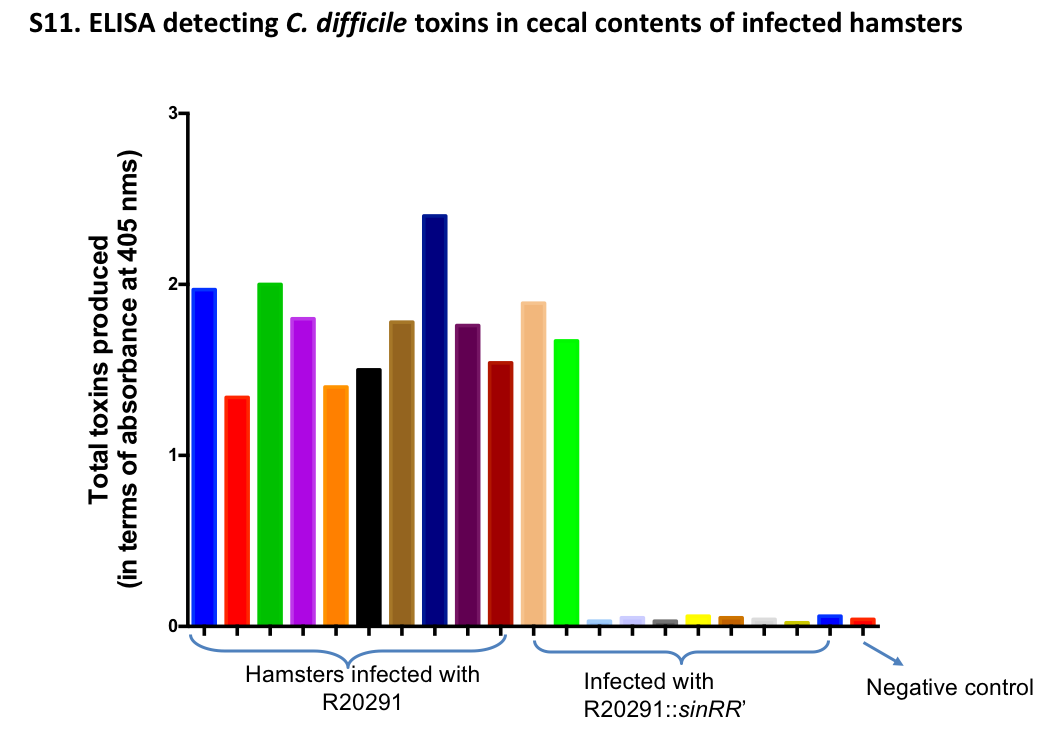

Supplement: S11 Fig — Cecal contents harvested upon post-mortem were analyzed using C. difficile premier Toxin A &B ELISA kit from Meridian Diagnostics Inc. (Cincinnati, OH), following manufacturer’s instruction. Negative control from the ELISA kit used along with the test samples. Each bar represents one animal. (TIF) [file ppat.1006940.s011.tif]

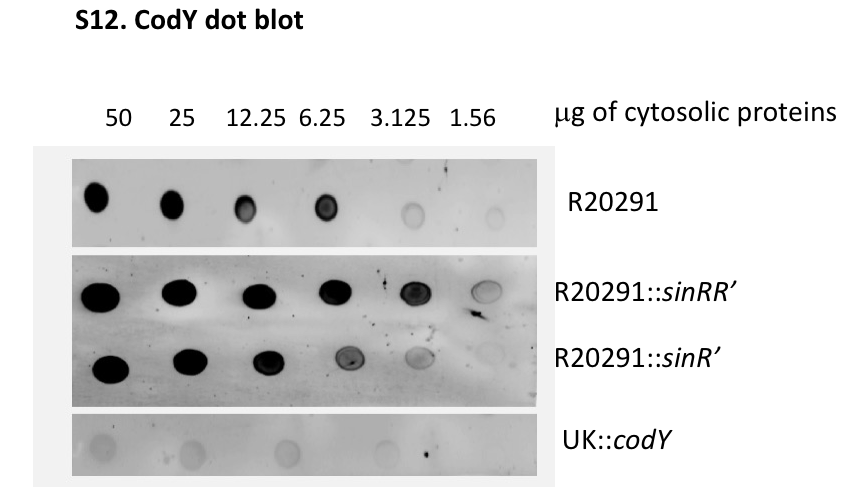

Supplement: S12 Fig — UK::codY mutant was used as a control. (TIF) [file ppat.1006940.s012.tif]
