# Supplementary material for: Pleiotropic roles of Clostridium difficile sin locus
Source: PLoS Pathog. 2018 Mar 12;14(3):e1006940. doi: 10.1371/journal.ppat.1006940 (PMC5864091; doi:10.1371/journal.ppat.1006940)
Supplement: S1 Table — (DOCX) [file ppat.1006940.s013.docx]

| **Bacterial strain or plasmid** | **Relevant features or genotype** | **Source or reference** |
| --- | --- | --- |
| *Clostridium difficile* JIR8094 | Erm^s^ derivative of strain 630 | O’Connor *et al.* (2006) |
| *Clostridium difficile* R20291 | Clinical isolate - NAP1/027 ribotype, isolated in 2006 following an outbreak in Stoke Mandeville Hospital, UK | Stabler *et al.* (2009) |
| *Escherichia coli* DH5α | *endA1 recA1 deoR hsdR17 (r*_K_^−^ *m*_K_^+^*)* | NEB |
| *Escherichia coli* S17-1 | Strain with integrated RP4 conjugation transfer function; favors conjugation between *E. coli* and *C. difficile* | Teng *et al.* (1998) |
| *Clostridium difficile* JIR8094::*sinR* R” | JIR8094 with intron insertion within *sinR* | This study |
| *Clostridium difficile* R20291::*sinR*R’ | R20291with intron insertion within *sinR* | This study |
| *Clostridium difficile* R20291::*sinR’* : | R20291with intron insertion within *sinR’* | This study |
| *Clostridium difficile* UK1 | Clinical isolate | Sorg *et al*., (2010) |
| *Clostridium difficile UK1::codY* |  | Mooyottu *et al*. (2014) |
| *R20291::sigD* | R20291with intron insertion within *sigD* | This study |
| pMTL007-CE5 | ClosTron plasmid | Heap *et al.* (2010) |
| pMTL007-CE5:Cdi-*sin*R-141a | pMTL007-CE5 with group II intron targeted to *sinR* | This study |
| pMTL007-CE5:Cdi-*sin*R’-a | pMTL007-CE5 with group II intron targeted to *sinR’* | This study |
| pMTL007-CE5:Cdi-*sigD* | pMTL007-CE5 with group II intron targeted to *sigD* | This study |
| pRPF185 | *E. coli*/*C. difficile* shuttle plasmid | Fagan *et al*. (2011) |
| pRG300 | pRPF185 containing *sinR* under inducible *tet* promoter | This study |
| pRG334 | pRPF185 containing *sinRR’* under inducible tet promoter | This study |
| pRG306 | pRPF185 containing *sinR’* under inducible *tet* promoter | This study |
| pRG291 | pRPF185 containing *sigD* under inducible *tet* promoter | This study |
| pRG310 | pMTL84151 containing 759bp upstream *sinR* with *sinR* gene (*sinR* under own promoter) | This study |
| pRG311 | pMTL84151 containing 759bp upstream *sinR* with *sinRR’* gene (*sinRR’* under own promoter) | This study |
| pRG312 | pMTL84151 containing 300bp upstream *spo0A* with *spo0A* gene | This study |
| pRG329 | pET16B containing *sinR* gene with His tag | This study |
| pRG327 | pET16B containing *sinR’* gene with His tag | This study |
| pRG359 | pET16B containing *codY* gene with His tag | This study |
| pGST parallel II | GST parallel II vector for GST fusions | Sheffield *et al.* (1999) |
| pRG331 | GST parallel II containing *sinR’* with GST tag | This study |

References:

1. O'Connor JR, Lyras D, Farrow KA, Adams V, Powell DR, Hinds J, et al. Construction and analysis of chromosomal *Clostridium difficile* mutants. Mol Microbiol. 2006;61(5):1335-51. doi: 10.1111/j.1365-2958.2006.05315.x. PMID: 16925561.
2. Stabler RA, He M, Dawson L, Martin M, Valiente E, Corton C, et al. Comparative genome and phenotypic analysis of *Clostridium difficile* 027 strains provides insight into the evolution of a hypervirulent bacterium. Genome Biol. 2009;10(9):R102. doi: 10.1186/gb-2009-10-9-r102. PMID: 19781061.
3. Teng F, Murray BE, Weinstock GM. Conjugal transfer of plasmid DNA from *Escherichia coli* to enterococci: a method to make insertion mutations. Plasmid. 1998;39(3):182-6. doi: 10.1006/plas.1998.1336. PMID: 9571134.
4. Sorg JA, Sonenshein AL. Inhibiting the initiation of *Clostridium difficile* spore germination using analogs of chenodeoxycholic acid, a bile acid. J Bacteriol. 2010;192(19):4983-90. doi: 10.1128/JB.00610-10. PMID: 20675492.
5. Mooyottu S, Kollanoor-Johny A, Flock G, Bouillaut L, Upadhyay A, Sonenshein AL, et al. Carvacrol and trans-cinnamaldehyde reduce *Clostridium difficile* toxin production and cytotoxicity *in vitro*. Int J Mol Sci. 2014;15(3):4415-30. doi: 10.3390/ijms15034415. PMID: 24625665.
6. Heap JT, Kuehne SA, Ehsaan M, Cartman ST, Cooksley CM, Scott JC, et al. The ClosTron: Mutagenesis in *Clostridium* refined and streamlined. J Microbiol Methods. 2010;80(1):49-55. doi: 10.1016/j.mimet.2009.10.018. PMID: 19891996.
7. Fagan RP, Fairweather NF. *Clostridium difficile* has two parallel and essential Sec secretion systems. J Biol Chem. 2011;286(31):27483-93. doi: 10.1074/jbc.M111.263889. PMID: 21659510.
8. Sheffield P, Garrard S, Derewenda Z. Overcoming expression and purification problems of RhoGDI using a family of "parallel" expression vectors. Protein Expr Purif. 1999;15(1):34-9. doi: 10.1006/prep.1998.1003. PMID: 10024467.
