# Supplementary material for: Pleiotropic roles of Clostridium difficile sin locus
Source: PLoS Pathog. 2018 Mar 12;14(3):e1006940. doi: 10.1371/journal.ppat.1006940 (PMC5864091; doi:10.1371/journal.ppat.1006940)
Supplement: S2 Table — (DOCX) [file ppat.1006940.s014.docx]

**S2 Table. Oligonucleotides used in this study**

| Name | Sequence (5’ 🡪 3’) | Description |
| --- | --- | --- |
| EBS-U | CGA AAT TAG AAA CTT GCG TTC AGT AAAC | Group II intron specific primer |
| ORG522 | CTAAAGTGGAGGGATAATAATTGGCAAATATAGG | *sinR*-Forward |
| ORG523 | TTATATTTCTTTGTATTTAATGATGCAC | *sinR*-Reverse |
| ORG549 | GTTAACAGATCTGAGCTCCTAAAGTGGAGGGATAATAATTG | *sinR*-Forward with SacI-pRPF185 (pRG300) cloning |
| ORG550 | AAGTTTTATTAAAACTTATAGGATCCTTATATTTCTTTGTATTTAATGATGCAC | *sinR*-Reverse with BamH1*-*pRPF185 (pRG300) cloning |
| ORG551 | GTTAACAGATCTGAGCTCCTGTAATAAGAAGATGTTTTTTAATGG | *spo0A*-Forward with SacI*-*pRPF185 (pRG301) cloning |
| ORG552 | AAGTTTTATTAAAACTTATAGGATCCTTATTTAACCATACTATGTTCTAGTC | *spo0A*-Reverse with BamH1*-*pRPF185 (pRG301) cloning |
| ORG536 | AGCGTTAACAGATCTGAGCTCGGAGGCGTAGTTAATGAATAG | *sigD*-Forward with SacI-pRPF185 (pRG291) cloning |
| ORG531 | AAGTTTTATTAAAACTTATAGGATCCCTATATAGAATATTTAAGTTCTTTTATCTTGTTTC | *sigD*-Reverse with BamH1-pRPF185 (pRG291) cloning |
| ORG553 | GTTAACAGATCTGAGCTCGTGAGGGAAATAGTAACAATAATGAATTATATAG | *sigR’* Forward with SacI-pRPF185 (pRG306) cloning |
| ORG554 | AAGTTTTATTAAAACTTATAGGATCCTTATATTTTATTCTTTTTTATGATGTCTATAATC | *sigR’* Reverse with BamH1 *-*pRPF185 (pRG306) cloning |
| ORG555 | CTTCTTATTTTTATGGTACCATGTAATATCACCCTCTTTAAAAATTTTTTTATTATTATATC | *sinR*-Forward with *Kpn1* –pMTL84151 (pRG310 cloning) |
| ORG556 | GGGCATCGAAATAAAAAACTAGTTTATATTTCTTTGTATTTAATGATGCAC | *sinR-*Reverse with *EcoR1-*pMTL84151 (pRG310 cloning) |
| ORG557 | GGGCATCGAAATAAAAAACTAGTTTATATTTTATTCTTTTTTATGATGTCTATAATCTG | *sinR’-*Reverse with *EcoR1-*pMTL84151 (pRG311cloning) |
| ORG559 | CTTCTTATTTTTATGGTACCGGTGCAATAACTCATGTTTTTAG | *spo0A* upstream + *spo0A-*Forward with *Kpn1*–pMTL84151 (pRG312) cloning |
| ORG560 | GGGCATCGAAATAAAAAACTAGTGACTCTCATATTTAAACCTCCAC | *spo0A* reverse with *EcoR1*–pMTL84151 (pRG312 cloning) |
| ORG582 | CTCGAGTTGGCAAATATAGGAAAAATAATAGG | *sinR* forward with *XhoI* to clone in pET16B |
| ORG583 | GGATCCTTAGTGGTGATGGTGATGATGTATTTCTTTGTATTTAATGATGCAC | *sinR* reverse with *BamH1* to clone in pET16B |
| ORG584 | CTCGAGATGAATTATATAGGTAAAAGAC | *sinR’* forward with *XhoI* to clone in pET16B |
| ORG585 | GGATCCTTAGTGGTGATGGTGATGATGTATTTTATTCTTTTTTATGATGTC | *sinR’* reverse with *BamH1* to clone in pET16B |
| ORG-19 | CGATACGACCGAAAACCTGTATTTTCAGGGCGCCATGGGGATGAATTATATAGG | *sinR’* forward GST with *NcoI* to clone in GST-parallel-II vector |
| ORG620 | GCGGCCGCACTAGTTGAGCTCGTCGACTTATATTTTATTCTTTTTTATGATGT | *sinR’* reverse GST with *SalI* to clone in GST-parallel-II vector |
| ORG629 | TGGAATAAGCCAACAGGAGAGTTTTGTGAG | *codY* upstream-forward; for SinR binding experiment |
| ORG630 | GTAATCTTCCATGTTCTTCAGGAAAGATTT | *codY* upstream- revers; for SinR binding experiment |
| ORG72 | TAAAAAATAAACTGAGAAAATGATATACTAATTT | *gluD* upstream-Forward; for SinR binding (control) |
| ORG73 | TATAAATACGTTATAATTATGTATACTCCATT | *gluD* upstream-Reverse; for SinR binding (control) |
| ORG721 | TAAAATAGAAAATTT TTTTAATTTTCAAAATATATTCTACATATCTAATATGTAATTAC | *sin* locus upstream- with potential CodY binding sequence |
| ORG722 | GTAATTACATATTAGATATGTAGAATATATTTTGAAAATTAAAAAAATTTTCTATTTTA | *sin* locus upstream- with potential CodY binding sequence (complementary to ORG721) |
| ORG719 | TAGTTATAACTTCAAAAAAGACTGAAAATTAAGAAAAAAGAAATATAAAT | *tcdR* upstream- with CodY binding sequence |
| ORG720 | ATTTATATTTCTTTTTTCTTAATTTTCAGTCTTTTTTGAAGTTATAACTA | *tcdR* upstream-with CodY binding sequence-complementary to ORG719 |
| ORG702 | GTAATATATCCGATTTTAGCATATGCTAAAATATCATACATCAAATTTTTAACTACTTAC | Non- specific DNA sequence used for CodY binding (non-specific control) |
| ORG723 | GTAAGTAGTTAAAAATTTGATGTATGATATTTTAGCATATGCTAAAATCGGATATATTAC | Complementary to ORG702 used to generate dsDNA probe (non-specific control) |
