# Supplementary material for: Pleiotropic roles of Clostridium difficile sin locus
Source: PLoS Pathog. 2018 Mar 12;14(3):e1006940. doi: 10.1371/journal.ppat.1006940 (PMC5864091; doi:10.1371/journal.ppat.1006940)
Supplement: S3 Table — (DOCX) [file ppat.1006940.s015.docx]

S3 Table. Oligonucleotides used for QRT-PCR

| Primer | Sequence (5’ 🡪 3’) | Gene target |
| --- | --- | --- |
| RG-RT23 (F) | GAGGAGAGTGGAATTCCTAGTGTAG | *16srRNA* |
| RG-RT24 (R) | GGACTACCAGGGTATCTAATCCTGT | *16srRNA* |
| RG-RT25 (F) | AGGCAGGTTTACATCCAACATA | *sinR* |
| RG-RT26 (R) | AGTGGTATGTCTAAAGCAGTAGC | *sinR* |
| RG-RT27 (F) | AAAGACTTAAAGAAGAACGGAAAA | *sinR’* |
| RG-RT28 (R) | TTGGATTCTTTTTACCACTTTCG | *sinR’* |
| RG-RT29 (F) | AAGTTAATATTTTTGAAGATGAGGATG | CD630_2216 |
| RG-RT30 (R) | TGGGATACTTATTTTCACTTAACCAA | CD630_2216 |
| RG-RT33 (F) | CATGAAATAGGAGTACCAGCTCA | *spo0A* |
| RG-RT34 (R) | CTCCATGCAACCTCTATTGC | *spo0A* |
| RG-RT35 (F) | TGCTCATGTATTATGGCTGGTATTT | *murG* |
| RG-RT36 (R) | AGGTTTGGAGCAATCAAGAAAGA | *murG* |
| RG-RT37 (F) | TGACTTTACACTTTCATCTGTTTCTAGC | *sigE* |
| RG-RT38 (R) | GGGCAAATATACTTCCTCCTCCAT | *sigE* |
| RG-RT39 (F) | TGTTTATAGATACTGGGCTCTCTGG | *spoIID* |
| RG-RT40 (R) | GACTTGCTCTGTGTTAGTTCCATC | *spoIID* |
| RG-RT41 (F) | CGCTCCTAACTAGACCTAAATTGC | *sigF* |
| RG-RT42 (R) | GGAAGTAACTGTTGCCAGAGAAGA | *sigF* |
| RG-RT43 (F) | CCTCAGTGAAATCATCATCATAGTCTTTA | *gpr* |
| RG-RT44 (R) | CCTGGTAATTGGTCTTGGAAATAGA | *gpr* |
| RG-RT45 (F) | CAAACTGTTGTCTGGCTTCTTC | *sigG* |
| RG-RT46 (R) | GTGGTGTTAATACATCAGAACTTCC | *sigG* |
| RG-RT49 (F) | CATATGTTGCTAATCGAGTTCCTTTAT | *sigK* |
| RG-RT50 (R) | TCAACGGAAGATCAGGATGATTTA | *sigK* |
| sleB-RT (F) | GATATTGTAGAGAACCCCTAATCC | *sleB* |
| sleB-RT (R) | GCAAATCCTAAAGCTAAAAATAC | sleB |
| Gpr-RT (F) | GGTGTTACTATTAAGTTCTTGTCAT | *gpr* |
| Gpr-RT (R) | CTGGTGGAGGTGTTGGCAATACTAG | *gpr* |
| sspA-RT (F) | CTATCTGTTGCTTTTTCCAGCC | *sspA* |
| sspA-RT (R) | GTATGAGTAATTATCAACAAGTTG | *sspA* |
| spoVAC RT(F) | GTAGACCAAATAAGCCCAAAACC | *spoVAC* |
| spoVAC-(R) | CAGAACTAGCACCTAGTTTATC | *spoVAC* |
| spoVAD-(F) | gtggcgatttaataaatcaaatag | *spoVAD* |
| spoVAD- (R) | cacttcctgctcctgtaactgtcc | *spoVAD* |
| cdeC-RT (F) | GATGAAATAAATTCAGAAGACATGA | *cdeC* |
| cdeC-RT (R) | GGCACTGCATTTGATACAGAGAAG | *cdeC* |
| sleC-RT (F) | CTGTTCCATAGATACCATCTTC | *sleC* |
| sleC-RT (R) | GGGCAGTAAAGACTTAGGTGACC | *sleC* |
| cotCB-RT (F) | ggtacagaggaaatggctcatgttg | *cotCB* |
| cotCB-RT (R) | cttgtagtaaagtttactccattag | *cotCB* |
| cotE-RT (F) | GAATATTGATAAAGCATCATCATATG | *cotE* |
| cotE-RT (R) | GCCATAAGAGATGTTATAGGGGATG | *cotE* |
| cotB-RT (F) | GATTTTATCTTACACTGTTCTATTCC | *cotB* |
| cotB-RT (R) | GGACCATATTATGATGGAACATGCTC | *cotB* |
| cotA-RT (F) | CTTACCTAGAACTTCAACACCAGTTA | *cotA* |
| cotA-RT (R) | CATTGTGTAATCTTAAAGCTGTTGC | *cotA* |
| pdaA-RT (F) | CATCTAATATCTCAGTATTTGTTG | *pdaA* |
| pdaA-RT (R) | GCGAACAATCTTTAAAATATACACAA | *pdaA* |
| RG-RT3 (F) | TAATAAAAATACTGCCCTCGACAAA | *tcdA* |
| RG-RT4 (R) | ATAAATTGCATGTTGCTTCATAACT | *tcdA* |
| RG-RT7 (F) | CTGGACAATGGAAGGTGGTT | *tcdB* |
| RG-RT8 (R) | TTGATGGTGCTGAAAAGAAGTG | *tcdB* |
| RG-RT5 (F) | AACATCTTGGAATATCTGAATTTTTCTCTA | *tcdE* |
| RG-RT6 (R) | TCTGTCATTGCATCTAGTAAAATTGCT | *tcdE* |
| RG-RT1 (F) | CAAGAAATAACTCAGTAGATGATTTGCAA | *tcdR* |
| RG-RT2 (R) | TCTCCCTCTTCATAATGTAAAACTCTACTA | *tcdR* |
| codY-RT(F) | CACTCTGTCCTACTCCGAGC | *codY* |
| codY-RT(R) | TCTATCACACCTGCACTCTCA | *codY* |
| dccA-RT(F) | TCAATTACTGACCCATTAAC | *dccA* |
| dccA-RT(R) | CTCCTACATTATGACCTTCA | *dccA* |
| sigD-RT(F) | \| TGATAGAGAAGAGGAAGCTCCA \| \| --- \| | *sigD* |
| sigD-RT(R) | TCTGAAACACCTAGCACTTTTCC | *sigD* |
| ccpA-RT(F) | AATACCTCATGCGGCTTTTG | *ccpA* |
| ccpA-RT(R) | TTGCTACTGCTCCCATATCGT | *ccpA* |
