# Supplementary material for: Pleiotropic roles of Clostridium difficile sin locus
Source: PLoS Pathog. 2018 Mar 12;14(3):e1006940. doi: 10.1371/journal.ppat.1006940 (PMC5864091; doi:10.1371/journal.ppat.1006940)
Supplement: S8 Table — (DOCX) [file ppat.1006940.s020.docx]

| **Gene** | **R20291/ R20291::*sinRR’*** | | | |
| --- | --- | --- | --- | --- |
|  | **Fold change (12h )** | **adj. *p* value** | **Fold change (16h)** | **adj. *p* value** |
| *sinR* | 4.578 | 0.0493E-06 | 6.975 | 1.486E-09 |
| *sinR’* | 6.893 | 1.41335E-10 | 10.456 | 0.54565E-03 |
| *spo0A* | 5.567 | 2.34045E-08 | 8.345 | 6.70941E-08 |
| *murG* | 4.342 | 0.00277972 | 5.234 | 1.07420E-01 |
| *sigE* | 5.234 | 0.008494963 | 25.35 | 3.44231E-05 |
| *spoIID* | 95.343 | 1.3567E-12 | 134.67 | 0.0045627 |
| *sigF* | 3.456 | 0.000688614 | 12.45 | 8.94523E-10 |
| *gpr* | 2.309 | 0.002356 | 5.54 | 1.28109E-02 |
| *sigG* | 10.35 | 0.003505323 | 24.56 | 2.3947E-08 |
| *sigK* | 4.56 | 4.28126E-10 | 15.09 | 9.42895E-07 |
| *sleB* | 2.39 | 3.8754E-3 | 4.67 | 0.0038671 |
| *sspA* | 54.24 | 2.9456E-11 | 78.24 | 5.49240E-03 |
| *spoVAC* | 245.986 | 0.005417615 | 689.78 | 0.98289E-09 |
| *spoVAD* | 134.734 | 0.000195106 | 276.28 | 0.00679831 |
| *cdeC* | 6.395 | 3.65845E-05 | 8.82 | 1.39475E-03 |
| *sleC* | 102.96 | 0.002345 | 240.45 | 7.39640E-04 |
| *cotCB* | 34.09 | 1.1946E-09 | 56.68 | 0.92587E-12 |
| *cotE* | 14.212 | 7.12107E-05 | 64.20 | 3.56729E-04 |
| *cotB* | 121.39 | 4.77323E-05 | 300.57 | 2.09561E-12 |
| *cotA* | 23.556 | 0.0056734 | 39.51 | 2.78457E-07 |
| *pdaA* | 2.389 | 0.003080399 | 19.83 | 6.27361E-03 |
| CDR20291_2213 | 1.1253 | 3.52950E-03 | 0.946 | 4.98451E-05 |

**S8. Table. QRT-PCR analysis of selected genes in *sinRR’* mutants**
