# Supplementary material for: Pleiotropic roles of Clostridium difficile sin locus
Source: PLoS Pathog. 2018 Mar 12;14(3):e1006940. doi: 10.1371/journal.ppat.1006940 (PMC5864091; doi:10.1371/journal.ppat.1006940)
Supplement: S1 Text — (DOCX) [file ppat.1006940.s021.docx]

**Plasmid construction:**

**pRG291:** A 670bp fragment containing *sigD* was amplified using primers org536 and org531 and cloned as *BamH1/Sac1* into pRPF185.

**pRG300:** A 339bp fragment containing *sinR* was amplified using primers org549 and org550 and cloned as *BamH1/Sac1* into pRPF185.

**pRG306:** A 315bp fragment containing *sinR’* was amplified using primers org553 and org554 and cloned as *BamH1/Sac1* into pRPF185.

**pRG307:** A 1025bp fragment containing region upstream of *sinR* with *sinR* was amplified using primers org555 and org556 and cloned into the MCS of pGEM-T

**pRG308:** A 1364bp fragment containing region upstream of *sinR* with *sinRR’* was amplified using primers org555 and org557 and cloned into the MCS of pGEM-T

**pRG309:** A 1465bp fragment containing region upstream of *spo0A* with *spo0A* was amplified using primers org559 and org560 and cloned into the MCS of pGEM-T

**pRG310:** The 1025bp upstream *sinR* with *sinR* sequence from pRG307 was cloned as *EcoR1/Kpn1* into pMTL84151.

**pRG311:** The 1364bp upstream *sinR* with *sinRR’* sequence from pRG308 was cloned as *EcoR1/Kpn1* into pMTL84151.

**pRG312:** The 1465bp upstream *spo0A* with *spo0A* sequence from pRG309 was cloned as *EcoR1/Kpn1* into pMTL84151.

**pRG324:** A 363bp fragment containing *sinR* with C term His tag was amplified using primers org582 and org583 and cloned into the MCS of pGEM-T

**pRG325:** A 339bp fragment containing *sinR’* with C term His tag was amplified using primers org584 and org585 and cloned into the MCS of pGEM-T.

**pRG327:** The 339bp *sinR’* sequence with 6X His tag from pRG325 was cloned as *Xho1/BamH1* into pET16B for purification.

**pRG329:** The 363bp *sinR* sequence with 6X His tag from pRG324 was cloned as *Xho1/BamH1* into pET16B for purification.

**pRG330:** A 315bp fragment containing *sinR’* was amplified using primers org619 and org620 and cloned into the MCS of pGEM-T.

**pRG331:** A 315bp *sinR’* sequence from pRG330 was cloned as *Nco1/Sal1* into GST parallel II yielding *sinR’* in-frame with GST tag for GST pull down experiment.

**pRG334:** A 654bp fragment containing *sinRR’* was amplified using primers org549 and org554 and cloned as *BamH1/Sac1* into pRPF185.

**pRG359:** The 786bp *codY* sequence with 6X His tag was amplified using primers org 675 and org676 and cloned as *Xho1/BamH1* into pET16B for purification.

**pMTL007-CE5:Cdi-*sin*R-141a, pMTL007-CE5:Cdi-*sin*R’-129s, pMTL007-CE5:Cdi-*sigD*:** Introns specific for *sinR*, *sinR’* and *sigD* were designed following the Perutka algorithm at ClosTron.com. The introns were synthesized and cloned inbetween *BsrGI* and *HindIII* sites in pMTL007-CE5 vector.
