# Supplementary material for: Pleiotropic roles of Clostridium difficile sin locus
Source: PLoS Pathog. 2018 Mar 12;14(3):e1006940. doi: 10.1371/journal.ppat.1006940 (PMC5864091; doi:10.1371/journal.ppat.1006940)
Supplement: S2 Text — (DOCX) [file ppat.1006940.s022.docx]

**Supplemental Methods:**

**Quantitative reverse transcription PCR (qRT-PCR)**

*C. difficile* cultures were grown in TY medium for 12h and were used for total RNA extraction following a previously described protocol [22, 60, 72]. After DNase (Turbo; Ambion) treatment, a 30 µL reaction was set up with 5 µg of template RNA, 4 µL of deoxynucleoside triphosphates (dNTP; 10mM each), 1 µg of hexamer oligonucleotide primer (5 µg/µL pdN_6_; Roche), and 6 µL of reverse transcription (RT) buffer and was heated at 80 °C for 5 min and cDNA was synthesized at 42 °C for 2 hours using avian myeloblastosis virus (AMV) reverse transcriptase (Promega). Final 20 µL reaction volume containing 10 ng of cDNA, 400 nM gene-specific primers, and 12.75 µL of SYBR PCR master mix (BioRad) was used to perform Real-time quantitative PCR using iQPCR real-time PCR instrument (BioRad). Quantity of cDNA of a gene in each sample was normalized to the quantity of *C. difficile* 16S rRNA gene and the ratio of normalized target concentrations (threshold cycle [2^−ΔΔCt^] method) [22, 60, 72] gives the relative change in gene expression. A minimum of three biological replicates were used per sample.

**Quantification of c-di-GMP in *C. difficile* by High Performance Liquid Chromatography (HPLC).**

Nucleotides were extracted and quantified from *C. difficile* as previously described with some modifications [61]. Briefly, cells from 50ml of early stationary phase cultures of R20291and R20291::*sinRR’* strains were harvested by centrifugation at 3000g for 20 min. The optical densities of the cultures at the time of harvest was recorded and dilutions were plated to determine the number of CFU extracted. The bacterial cell pellet was washed in 1ml of TE buffer (10mM Tris (pH7.5), 1mM EDTA, pH8) and vortexed in 500 μl of nucleotide extraction buffer (40% acetonitrile 40% methanol in 0.1N formic acid)) and incubated at −20°C for 30 min. Samples were centrifuged for 5 min at 17,000 × *g* in 4°C, and the supernatant was immediately neutralized by adding 4 μl of 15% (wt/vol) NH_4_HCO_3_ per 100 ul of sample_._ One in ten dilution of the samples were made with deionized water and 50 μl of the sample aliquots was injected into high performance liquid chromatography (HPLC, Schimadzu Corp. Nakagyo-ku, Kyoto, Japan) and the analytes were separated on C18 reverse phase HPLC column (4.6 by 250 mm; 5 μm) with the following gradient of solvent A (10 mM ammonium acetate in water) to solvent B (0.1% TFA v/v acetonitrile). The following elution method was used: 0 to 1 min, isocratic hold at 0% (of eluent B); 1 to 2 min, 0 to 20% linear gradient; 2 to 4 min, isocratic hold at 20%; 4 to 5 min, 20 to 40% linear gradient; 5 to 7 min, isocratic hold at 40%; 7 to 8, 40 to 70% linear gradient; 8 to 10 min, isocratic hold at 70%; 10 to 11, 70 to 90% linear gradient; 11 to 13 min, isocratic hold at 90%; 13 to 14 min, 90 to 0% linear gradient; 14 to 16 min, 0% isocratic hold to re-equilibrate the column for the next sample injection (16 min total). The c-di-GMP was determined by fitting the peak area to a linear standard curve obtained by analyzing serial dilutions (250 nM, 125 nM, 62.5 nM, 31.25 nM, 15.625 nM, and 7.8125 nM) of standard c-di-GMP (Sigma-Aldrich, Inc. St. Louis, MO, USA) using the same HPLC method. The intracellular levels of c-di-GMP was calculated by dividing the total amount of c-di-GMP extracted by the total intracellular volume of bacteria extracted. The later was determined by multiplying the number of cells extracted, based on CFU counts by the volume of one bacterial cell. To measure the volume of a single bacterial cell, average cell length and width were measured from the electron microscopic images of the R20291 and R20291::*sinRR’* mutant cells (at least fifty independent cells were measured) and were estimated to be 3.46×10^-16^ L using ImageJ software.
